# Thermoelectric Gating Organic Electrochemical Transistors Enabled by Printable Ionogels With n‐p Convertible Thermopower

**DOI:** 10.1002/advs.75450

**Published:** 2026-04-23

**Authors:** Xingyu Hu, Xinwen Yan, Ling Huang, Xiaohang Zhang, Xuan Cao, Cong Zhang, Qingqing Sun, Hanyu Jia, Xuying Liu

**Affiliations:** ^1^ School of Materials Science and Engineering Zhengzhou University Zhengzhou China; ^2^ Hubei R&D Center of Hyperbranched Polymers Synthesis and Applications Key Laboratory of Catalysis and Energy Materials Chemistry of Ministry of Education & Hubei Key Laboratory of Catalysis and Materials Science South‐Central Minzu University Wuhan China

**Keywords:** ionogels, n‐p conversion, organic electrochemical transistor, stretchable electronics, thermoelectric gating

## Abstract

Organic electrochemical transistors (OECTs) are promising for bioelectronics and low‐power logic owing to their mixed ionic‐electronic conduction and mechanical softness. However, thermoelectric gating of OECTs remains unexplored due to the incompatibility of rigid, electronically dominated thermoelectric modules with hydrated soft systems. Here, we introduce printable thermoelectric ionogels with n‐p convertible thermopower, where the Seebeck coefficient is tunable from –3.61 to +9.74 mV K^−^^1^ through facile [EMIm][Cl] doping. These ionogels act simultaneously as thermoelectric legs and ionic dielectrics, enabling direct integration with OECTs to realize soft, complementary p–n thermoelectric modules. We demonstrate thermoelectric gating of both BBL‐ and p(g2T‐T)‐based OECTs, achieving high on/off ratios (>10^3^) and robust transconductance under low temperature gradients (<30 K), while retaining mechanical stretchability. This strategy provides a general framework for coupling thermoelectric functionality with OECTs, opening new avenues for energy‐autonomous, flexible electronics with built‐in thermal sensing and adaptive control.

## Graphical abstract

A type of thermoelectric ionogels with convertible n‐p thermopowers are demonstrated with Seebeck coefficients ranging from ‐3.61 to +9.74 mV K^−1^. Thus, thermoelectric gating organic electrochemical transistor is achieved by employing ionogel as thermoelectric modules and ionic dielectrics simultaneously, paving the road to self‐powered, highly integrated, printing, and stretchable electronics.

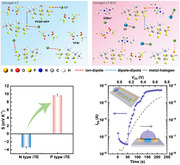

## 1 Introduction

Organic electrochemical transistors (OECTs) have emerged as a powerful class of devices for flexible and bio‐interfacing electronics, owing to their intrinsic compatibility with soft matter, low‐voltage operation, and mixed ionic‐electronic conduction. These attributes have enabled diverse applications in biochemical sensing, electrophysiological recording, neuromorphic computing, and wearable logic systems [1, 2, 3, 4, 5, 6, 7, 8]. Beyond conventional operation, researchers have developed various stimulus‐gated transistors (e.g., piezoelectric, triboelectric, and optically responsive devices) by incorporating functional layers or dopants within the channel region [9, 10, 11, 12, 13, 14, 15]. However, despite these advances, achieving autonomous and intelligent OECTs remain a significant challenge. In particular, their ability to respond directly to thermal stimuli is limited by the lack of efficient mechanisms for harvesting temperature gradients and converting them into reliable gate voltages. Furthermore, existing integration approaches often suffer from weak coupling, bulky geometries, and material incompatibilities, restricting the scalability and practicality of thermally gated OECTs.

Thermoelectric gating, in which a temperature gradient is converted into a gating voltage, offers a compelling strategy to overcome these limitations and enable self‐powered modulation [16, 17]. Yet, this approach faces critical obstacles at both the technological and materials levels. From an integration standpoint, serially connecting thermoelectric modules with transistors via wires results in inefficient signal transfer and limited modulation strength. While monolithic integration of OECTs with organic photovoltaics (OPVs) has been demonstrated through advanced patterning techniques [18], analogous integration with thermoelectric remains difficult, as effective thermal gradients typically require large device geometries. On the materials side, conventional thermoelectrics provide nonideal strategies: inorganic materials (e.g., bismuth telluride alloys) offer high conversion efficiency but suffer from poor solution processability and potential toxicity [19, 20, 21, 22], while organic materials, despite their flexibility and processability, generally exhibit low Seebeck coefficients (∼100 µV K^−^^1^), necessitating large‐scale thermocouple arrays with hundreds to thousands of legs for effective gating—dramatically increasing fabrication complexity [23, 24, 25, 26].

Ionic thermoelectric (*i*‐TE) materials provide a promising alternative. Their exceptionally high Seebeck coefficients, combined with excellent solution processability, environmental stability, and tunable electrochemical properties, make them attractive candidates for thermoelectric gating transistors [27, 28]. Recent studies on ionogels, such as PDMAA‐[EMIm][DCA]/Fe(II)‐Fe(III) (32.4 mV K^−^^1^) [29] and MXene‐enhanced MMA/MA/[EMIm][TFSI] (−8.8 mV K^−^^1^) [30], have demonstrated remarkable efficiency in harvesting low‐grade heat. Most reported *i*‐TEs still exhibit only one polarity of thermopower. Nevertheless, recent studies have started to explore strategies for achieving tunable p‐n behavior within a single ionogel system (such as phase separation, side‐chain engineering, and electrode engineering, Table S1), though related reports remain limited [31, 32, 33, 34, 35, 36]. This limitation poses a fundamental obstacle, as p‐n conversion is essential for constructing highly integrated *i*‐TE modules, analogous to the role of complementary p‐ and n‐type semiconductors in conventional thermoelectrics. Without matched p‐ and n‐type legs, it is impossible to form high‐output, series‐connected thermocouples that can maximize voltage generation and power density [37]. Moreover, the absence of controllable polarity restricts design flexibility, hinders module symmetry, and ultimately constrains the integration of *i*‐TEs with functional devices such as OECTs. Therefore, achieving reliable p‐n type switching in all‐solid‐state *i*‐TE materials is a critical step toward translating their extraordinary ionic Seebeck performance into practical, high‐performance energy‐harvesting and gating systems.

Here, we address this critical challenge by demonstrating a controllable conversion from n‐type (PVDF‐HFP/LiTFSI, Ionogel‐LT) to p‐type (PVDF‐HFP/LiTFSI/[EMIm][Cl], Ionogel‐LT‐ECl) characteristics in all‐solid‐state ionic thermoelectric materials. Molecular dynamics (MD) simulations combined with spectroscopic analysis reveal the underlying atomic‐scale mechanism: the polarity reversal of thermopower arises from the modulated ion‐polymer and ion‐ion interactions, where competitive coordination amplifies the migration disparity between anions and cations. Building upon this tunability, we integrate a photo patternable fluoropolymer insulating layer to fabricate inkjet‐printed, monolithically integrated, thermal‐gradient‐gated OECTs. These devices operate efficiently under small temperature gradients, thereby bridging the gap between high‐performance *i*‐TE modules and functional transistor platforms. Our findings not only establish a pathway to complementary p‐ and n‐type *i*‐TE legs for module‐level design but also open opportunities for self‐powered, scalable, and intelligent wearable electronics.

## 2 Results and Discussion

### 2.1 Thermoelectric Behaviors of n‐p Convertible Thermoelectric Ionogels

**FIGURE 1:**
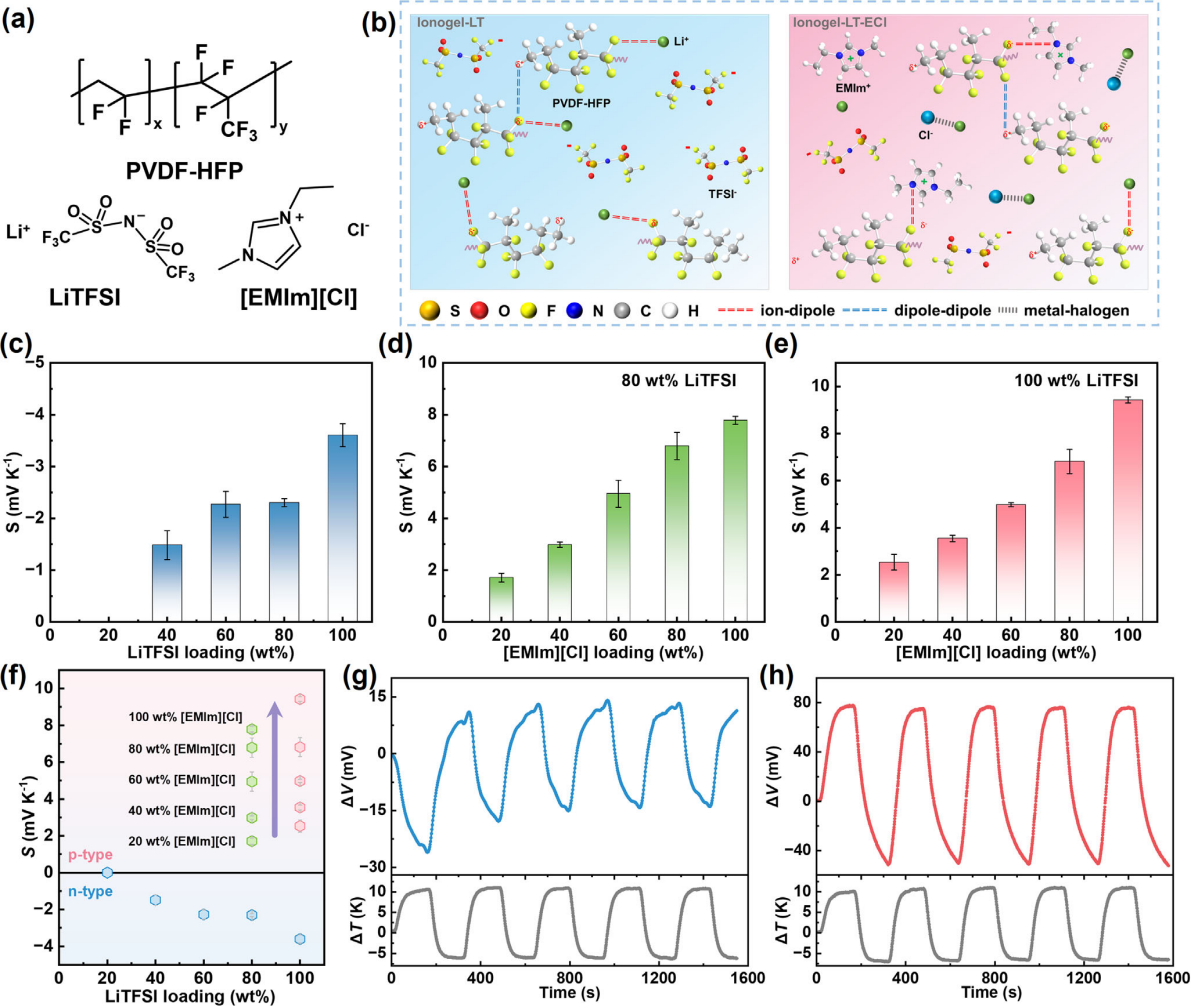
Thermoelectric ionogels with convertible n‐p thermopowers. (a) Molecular structure of Ionogel‐LT (n‐type) and Ionogel‐LT‐ECl (p‐type). (b) Schematic illustration of ion transport mechanism of Ionogel‐LT and Ionogel‐LT‐ECl. (c) Seebeck coefficients of Ionogel‐LT upon varied LiTFSI loading weights. Seebeck coefficients of Ionogel‐LT‐ECl with varied [EMIm][Cl] loading weights upon fixed LiTFSI loading of 80 wt.% (d) and 100 wt.% (e). (f) Tunable ionic thermopower of Ionogel‐LT‐ECl_x_. Typical ΔV‐ΔT curves of Ionogel‐LT(g) and Iongel‐LT‐ECl (h). All measurements were conducted at 40% relative humidity.

The flexible solid‐state thermoelectric ionogel, denoted Ionogel‐LT, was obtained via the evaporation of the N,N‐dimethylformamide (DMF) solution of poly(vinylidene fluoride‐co‐hexafluoropropylene) (PVDF‐HFP) and lithium bis(fluorosulfonyl)imide (LiTFSI), in which the weight ratios between PVDF‐HFP and LiTFSI range from 40 to 100 wt.% (Figure S1a). PVDF‐HFP exhibits a biphasic microstructure: crystalline PVDF‐rich phase providing mechanical robustness, and amorphous HFP‐rich phase that readily absorbs polar solvents, acting as plasticizing domains [38]. Within this matrix, LiTFSI serves as the ionic donor for n‐type conduction. The introduction of another ionic liquid, namely 1‐ethyl‐3‐methylimidazolium chloride ([EMIm][Cl]), transforms Ionogel‐LT into Ionogel‐LT‐ECl, reversing its thermopower polarity from n‐type to p‐type via competitive coordination. The changes of dominant ionic polarity in the thermoelectric ionogels result from the competitive mechanism in ion‐dipole and dipole‐dipole interactions (Figure 1b). In the n‐type Ionogel‐LT, the highly electronegative fluorine (F) atoms in PVDF‐HFP anchor Li^+^ via ion‐dipole interactions. Simultaneously, the bulky TFSI^−^ anion critically disrupts crystallization within the thermoelectric ionogel matrix, lowering the energy barrier for ion migration and enhancing overall ion transport efficiency. The disparity in ion migration rates under applied temperature difference, where TFSI^−^ outpaces Li^+^ toward the hot end to generate a net negative charge accumulation, endowing Ionogel‐LT with distinct n‐type ionic thermoelectric characteristics. However, introducing [EMIm][Cl] into Ionogel‐LT triggers a fundamental restructuring of ionic interactions. Stronger metal‐halogen interaction of Li^+^‐Cl^−^ impairs the previous existing ion‐dipole interaction of Li^+^‐F, thus inducing faster EMIm^+^ cation migration than TFSI^−^ considering its dipole‐dipole (F‐F) interaction with PVDF‐HFP.

The influence of LiTFSI loading on thermoelectric behavior of Ionogel‐LT was first investigated by a custom‐made thermoelectric testing system, including a pair of Peltier elements to control the temperature between cold and hot end (Figure S1). As shown in Figure 1c and Figure S2, Ionogel‐LTs with different LiTFSI loading exhibit typical n‐type thermoelectric characterisitics, and thermopower of Ionogel‐LTs increased from ‐1.48 ± 0.28 mV K^−1^ to ‐3.61 ± 0.22 mV K^−1^ when increasing LiTFSI loading from 40 wt.% to 100 wt.% (relative to PVDF‐HFP). Interestingly, the ionic conductivity of Ionogel‐LT was also improved from 4.5×10^−4^ mS cm^−1^ to 0.24 mS cm^−1^, which was attributed to increased ion concentration in the ionogel matrix (Figure S3a.c, Scheme S4). To fully evaluate the impact of [EMIm][Cl] doping, we systematically varied the [EMIm][Cl]‐to‐LiTFSI weight ratio while maintaining fixed LiTFSI loadings (Ionogel‐LT_0.8_ for 80 wt.% or Ionogel‐LT_1_ for 100 wt.% relative to PVDF‐HFP). Intriguingly, introducing 20 wt.% [EMIm][Cl] triggered the same polarity reversal from n‐type to p‐type thermoelectric behavior in both Ionogel‐LT_0.8_ and Ionogel‐LT_1_ (Figure 1d,e). Notably, the n‐p conversion seems more significant in Ionogel‐LT_1_ than Ionogel‐LT_0.8_ when doped with the same amount of [EMIm][Cl] (Figure 1f; Figures S4–S6). During the periodical heating‐cooling cycles, Ionogel‐LT with 100 wt.% LiTFSI loading exhibits outstanding thermoelectric stability (Figure 1g). Based on the above results, Ionogel‐LT with 100 wt.% LiTFSI loading was chosen as the optimal thermoelectric ionogel for the subsequent research. In concrete, the optimized p‐type thermopower of 9.74 mV K^−1^ and ionic conductivity of 1.21 mS cm^−1^ were achieved in Ionogel‐LT‐ECl with 100 wt.% [EMIm][Cl]loading (Figure 1e;Figure S3d), as well as its excellent thermoelectric operation stability (Figure 1h). Consequently, the ionic power factor (PF) of Ionogel‐LT‐ECl surged from 2.59 µW m^−1^ K^−2^ to 108.08 µW m^−1^ K^−2^ after increasing [EMIm][Cl] loading to 100 wt.%, exhibiting superior thermoelectric conversion efficiency and potential for gating application (Figure S7).

In order to further boost the performance of n‐type Ionogel‐LT and p‐type Ionogel‐LT‐ECl, the impact of electrode material on the thermoelectric properties of ionogels were carefully investigated. Considering the above thermoelectric tests were performed by silver electrodes, gold and copper electrodes were employed for the thermoelectric ionogels to obtain a fair comparison on thermoelectric performance, providing an experimental basis for the subsequent thermal gradient gated OECT integration. The results revealed that Ionogel‐LT using gold electrodes exhibits the lowest thermopower of ‐0.60 mV K^−1^, followed by those using copper electrodes at ‐2.94 mV K^−1^, while Ionogel‐LT employing silver electrodes demonstrated the highest thermopower of ‐3.26 mV K^−1^. A similar trend was observed in Ionogel‐LT‐ECl, where ionogels using gold and copper electrodes only yielded thermopower of 2.06 and 3.20 mV K^−1^, respectively. In contrast, Ionogel‐LT‐ECl employing silver electrodes achieved a significantly higher value of 9.74 mV K^−1^ (Figure S8). This electrode‐dependent thermopower is attributed to the interaction between the dominant transporting ions and the electrodes. First‐principles calculations reveal stronger adsorption of TFSI^−^ (n‐type) and EMIm^+^ (p‐type) on Au and Cu than on Ag (Figures S9–S12 and Tables S2 and S3). Consequently, silver was selected as the electrode material for the subsequent integration of thermoelectric modules with OECTs. Furthermore, to further enhance thermoelectric gating efficiency of ionogels, the crystallinity degree of PVDF‐HFP serving as the ionogel network was carefully investigated by modulating the HFP content of PVDF‐HFP. Notably, ionogels using PVDF‐HFP with 10% HFP content exhibited fast thermal equilibrium dynamics (in 150 s) than those using PVDF‐HFP with 18% HFP content thermal response, revealing lower crystallinity degree was strongly associated with longer thermal equilibration time (Figure S13). In addition, owing to the abundant noncovalent intermolecular interaction rather than chemical bonding within the ionogel matrix, both n‐type and p‐type thermoelectric ionogels exhibit outstanding recyclability for reconfigurable thermoelectrics. To present this reconfigurability, fragmented pieces of Ionogel‐LT and Ionogel‐LT‐ECl are redissolved in DMF until a transparent solution was formed. Subsequently, the ionogel can be regenerated through solvent evaporation led by thermal annealing process (Figure S14). Notably, the regenerated ionogels retain their thermoelectric properties, a feature that significantly enhances the potential for recyclable energy harvesting devices.

**FIGURE 2:**
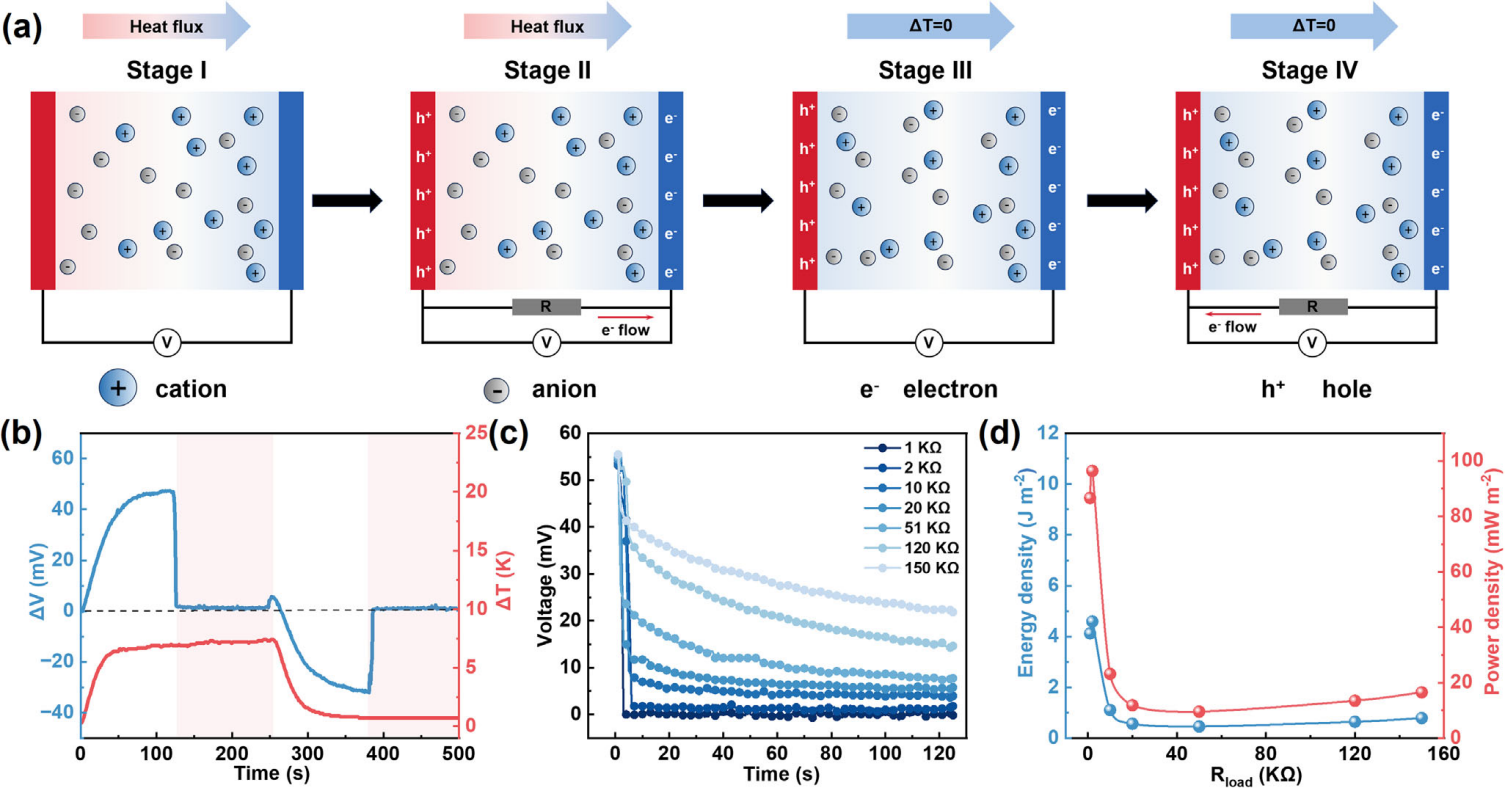
Mechanism and performance of Ionogel‐LT‐ECl based ionic thermoelectric capacitors (*i*‐TEC). (a) Working mechanism of Ionogel‐LT‐ECl based *i*‐TEC in a single thermocycle. (b) ΔV‐ΔT curve of the i‐TEC with an external load of 2 kΩ in a single thermocycle. (c) Output voltage decay curves under different external loads during stage II. (d) The energy density and power density of Ionogel‐LT‐ECl based *i*‐TEC upon different external loads.

Unlike conventional thermoelectric materials that rely on electrons and holes as charge carriers, thermoelectric ionogels based on the Soret effect cannot directly function as generators because ionic carriers cannot pass through the electrode interface into an external circuit. To achieve thermoelectric conversion, we assembled an ionic thermoelectric capacitor (*i*‐TEC) [39, 40, 41]. Given that Ionogel‐LT‐ECl with 100 wt.% [EMIm][Cl] loading exhibits the highest PF, it was selected as the active material to construct the *i*‐TEC. The voltage profile can be divided into four stages, as illustrated in the schematic diagram (Figure 2a). In Stage I, when a temperature gradient of 5 K is applied, cations primarily diffuse from the hot end to the cold end. Thus, the equilibrium of cation accumulation on the cold electrodes and anion accumulation on the hot electrodes was built, generating an open‐circuit voltage. In Stage II, an external load is connected to the *i*‐TEC, causing electrons to migrate through the external circuit between hot‐cold electrodes, thereby balancing the thermal voltage through ionic accumulation. As a result, the voltage rapidly decays to zero. Upon removing the ΔT and external load in Stage III, the ions accumulated at the electrodes revert to their original distribution. At the same time, electrons and holes remain on the electrodes, producing a reverse voltage. Finally, in Stage IV, when the external load is reconnected, electrons flow out through the external circuit, and the voltage drops to zero (Figure 2b). The output voltage across the external load depends on the resistance of the external load (R_load_), and decays slower during Stage II when the R_load_ is increased (Figure 2c). Therefore, the energy density achieved in a single thermocycle reaches a maximum of 10.52 J m^−2^ when connected with external load of 2 kΩ. Likewise, the average power density is maximized at 42.07 mW m^−2^ under the same external load condition of 2 kΩ (Figure 2d & Supporting Information for detailed calculation methods).

### 2.2 Mechanism of n‐p Conversion in Thermoelectric Ionogels

Optical characterization reveals that while Ionogel‐LT exhibits high transparency, introducing [EMIm][Cl] significantly reduces transmittance due to enhanced light scattering (Figures S15 and S16). Scanning electron microscopy (SEM) images also indicate negligible morphological differences between Ionogel‐LT and Ionogel‐LT‐ECl (Figure S17). Therefore, the polarity reversal of ionic thermopower in Ionogel‐LT after introducing [EMIm][Cl] can be ascribed to ion‐ion interactions rather than ion‐polymer interactions or morphological changes. Additionally, atomic force microscopy (AFM) analysis confirms both ionogels possess substantially increased surface roughness vs. pristine PVDF‐HFP (Figure S18), suggesting LiTFSI/[EMIm][Cl] incorporation creates percolated ion migration pathways within the polymer network. Energy‐dispersive X‐ray spectroscopy (EDS) mapping further confirms the uniform distribution of ion species in Ionogel‐LT and Ionogel‐LT‐ECl (Figure S19).

**FIGURE 3:**
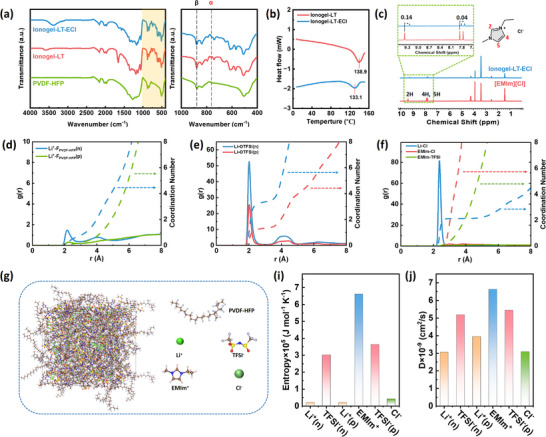
N‐p conversion mechanism of thermoelectric ionogels. (a) FTIR spectra of pristine PVDF‐HFP, Ionegel‐LT, and Ionogel‐LT‐ECl. (b) DSC curves of Ionogel‐LT and Ionogel‐LT‐ECl. (c) ^1^H‐NMR spectra revealing the chemical shift of H on imidazolium cations in Ionogel‐LT and Ionogel‐LT‐ECl. The calculated radial distribution function (RDF) and coordination number (CN) of Li^+^‐F_PVDF‐HFP_ (d), Li^+^‐O_TFSI‐_ (e) interaction in n‐type Ionogel‐LT and p‐type Ionogel‐LT‐ECl. (f) The calculated RDF and CN of Li^+^‐Cl^−^, EMIm^+^‐Cl^−^, and EMIm^+^‐TFSI^−^ pairs in p‐type Ionogel‐LT‐ECl. (g) Molecular dynamic (MD) snapshot of Ionogel‐LT‐ECl. The calculated entropy change (i) and diffusion coefficient change (j)of cations and anions in n‐type Ionogel‐LT and p‐type Ionogel‐LT‐ECl.

To elucidate the interactions between the polymer network and ions, Fourier‐transform infrared spectroscopy (FTIR) was conducted on Ionogel‐LT and Ionogel‐LT‐ECl in the wavenumber range of 4000–400 cm^−1^ (Figure 3a). The intensity of the crystalline phase (α‐phase) of PVDF‐HFP at 762 cm^−1^ (‐CH_2_ rocking) decreases when introducing LiTFSI, and disappears in Ionogel‐LT‐ECl when further introducing [EMIm][Cl]. Simultaneously, the amorphous phase (β‐phase) at 879 cm^−1^ becomes dominant, and the peak at 835 cm^−1^ gradually redshifts after the addition of ionic liquids, with another β‐phase peak emerging at 841 cm^−1^ in Ionogel‐LT‐ECl. Considering ion conduction in polymers primarily occurs in the amorphous regions, the increased β‐phase facilitates the ion migration of LiTFSI and [EMIm][Cl] across the polymer network. Furthermore, the formation of the β‐phase is associated with the interactions of ion‐polymer dipole moments, which can generate significant spontaneous polarization [33]. Additionally, differential scanning calorimetry (DSC) indicates the gel‐sol transition of Ionogel‐LT near 135°C, and the introduction of [EMIm][Cl] increases the integral area of the gel‐sol transition, which implies that more energy is required for this transition (Figure 3b). This results suggests that introducing [EMIm][Cl] enhances the Li^+^‐Cl^−^ interaction, thus EMIm^+^ transport (p‐type) becomes dominant in Ionogel‐LT‐ECl [42]. Nuclear magnetic resonance (NMR) results show that in Ionogel‐LT‐ECl, the signals for C^2^‐H, C^4^‐H, and C^5^‐H on the imidazolium ring shift upfield by 0.14 and 0.04 ppm, respectively [43]. These phenomena further indicate that the stronger interaction between Li^+^ and Cl^−^ effectively impairs hydrogen bonding interaction between C^2^‐H and Cl^−^ (Figure 3c) [25, 37].

To achieve comprehensive insights into the interactions between ions and the polymer and the entropy changes, molecular dynamics (MD) simulations were conducted for Ionogel‐LT and Ionogel‐LT‐ECl [44]. Details of the computational methods are provided in the Supporting Information. Figure 3g and Figure S20 present simulation snapshots of Ionogel‐LT and Ionogel‐LT‐ECl at atomic level, showing the uniform distribution of PVDF‐HFP, Li^+^, TFSI^−^, EMIm^+^, and Cl^−^ within the simulation box. The peak intensity of Li^+^ interacting with F atoms on the PVDF‐HFP chains at 2.18 Å weakens based on the radial distribution functions (RDFs), indicating that the partial ion‐dipole interactions between F atoms and Li^+^ cations are transformed into weaker forces in Ionogel‐LT‐ECl (Figure 3d). In Ionogel‐LT, a sharp peak at 2.02 Å suggests that TFSI^−^ occupies the first coordination shell of Li^+^. The weakened peak of Li‐O_TFSI_^−^ in Ionogel‐LT‐ECl indicates that the addition of [EMIm][Cl] results in more freely mobile TFSI^−^ anions in Ionogel‐LT‐ECl (Figure 3e). Simultaneously, the Li^+^‐Cl^−^ pair exhibits a very sharp peak at 2.4 Å, while EMIm^+^‐Cl^−^ shows a minor peak. These results suggest that Li^+^ tends to coordinate with Cl^−^, thereby weakening the coordination of TFSI^−^. Additionally, the interactions between EMIm^+^ cations and TFSI^−^ anions are negligible (Figure 3f). Figure 3i illustrates the entropy changes of cations and anions in Ionogel‐LT and Ionogel‐LT‐ECl. The results indicate that the entropy change of Li^+^ in Ionogel‐LT‐ECl is greater than that in Ionogel‐LT, with the trend of entropy changes for each ion being EMIm^+^>TFSI^−^>Cl^−^>Li^+^. This suggests that the EMIm^+^ cation, with its higher entropy, plays a dominant role in contributing to the ionic thermopower. Moreover, the greater the entropy change of the cations, the larger the corresponding entropy difference under a ΔT, leading to a higher ionic thermopower. In summary, the EMIm^+^ cation, with its higher entropy, possesses a greater driving force, thereby facilitating the transition from n‐type to p‐type thermoelectric ionogel. Additionally, the diffusion coefficients (D) of each ion in Ionogel‐LT and Ionogel‐LT‐ECl are shown in Figure 3j and Figure S21. It is evident that in Ionogel‐LT, the diffusion coefficient of the TFSI^−^ anion is greater than that of the Li^+^ cation, indicating that it functions as an n‐type *i*‐TE gel. In Ionogel‐LT‐ECl, the D value of the TFSI^−^ anion increases, consistent with the release of more freely mobile TFSI^−^ anions after the addition of [EMIm][Cl].

### 2.3 All‐in‐One Thermoelectric Gating OECTs

**FIGURE 4:**
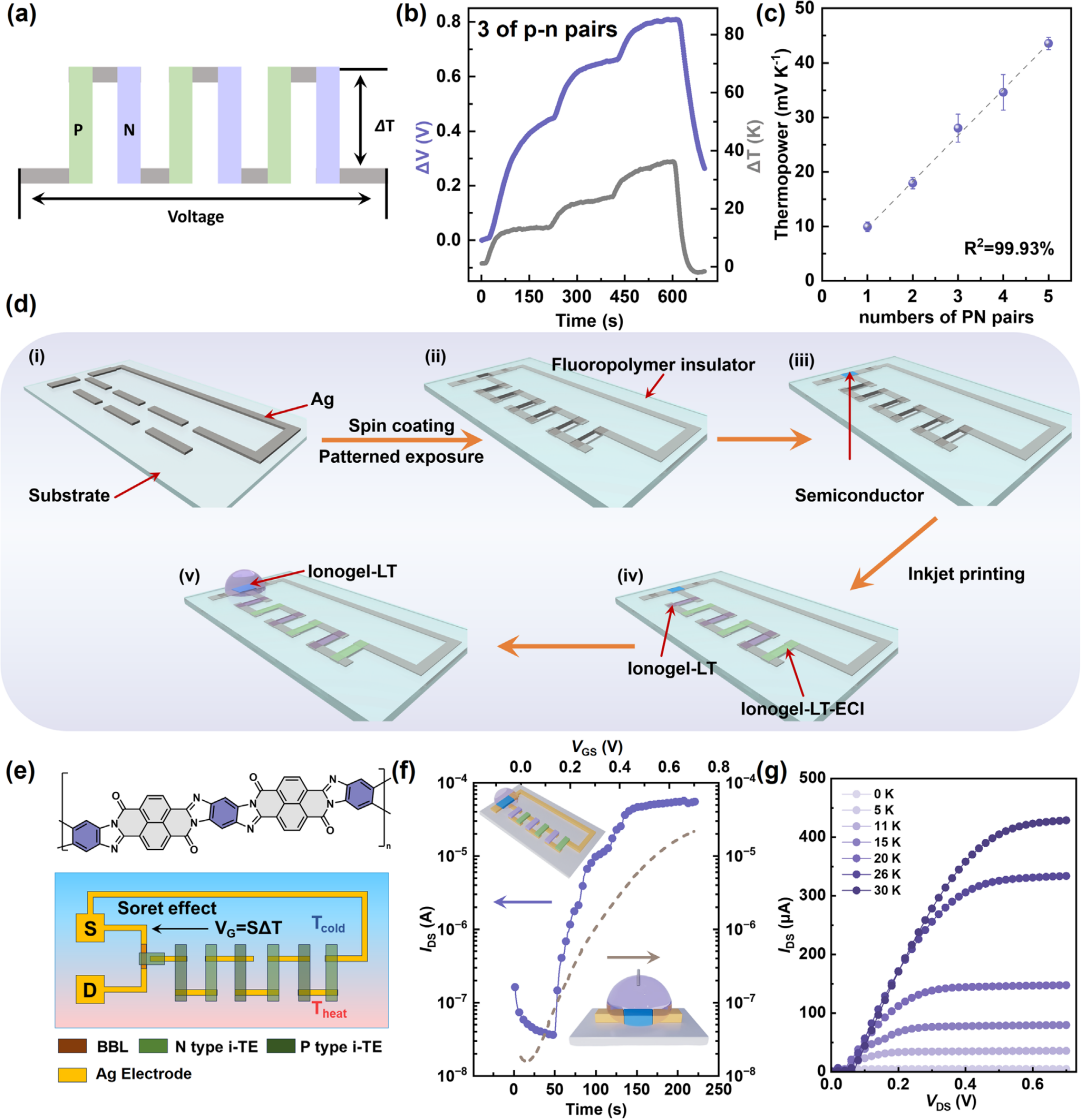
All‐in‐one thermoelectric gating OECTs. (a) Schematic illustration of integrated p‐n pairs based on thermoelectric ionogels. (b) Time‐dependent ΔT & ΔV curves of an *i*‐TE module consisting of three p‐n pairs. (c) The linear relationship between numbers of p‐n pairs and the output thermopowers. (d) Fabrication process flow diagram of the all‐in‐one thermoelectric gating OECT. (e) Schematic illustration of the device structure of thermoelectric gating OECT and the molecular structure of BBL for OECT channel. (f) Comparative transfer curves (channel length 50 µm, width 500 µm) of thermoelectric gating OECT and conventional OECT based on BBL channel upon applied gate voltage, and ΔT was set as 20 K. (g) Output curves of thermoelectric gating OECT upon different ΔT.

To obtain sufficient output Seebeck voltage, ionic thermoelectric module based on n‐type leg of Ionogel‐LT and p‐type leg of Ionogel‐LT‐ECl was constructed (Figure 4a). By altering the number of p‐n pairs connected in series, a significant thermal voltage was achieved (Figure S22). Specifically, three p‐n pairs of ionogel thermoelectric module output a Seebeck voltage of 0.8 V upon temperature gradient of 35 K (Figure 4b). A linear relationship between the number of p‐n pairs and the output Seebeck voltages was found with a fitting degree of 99.93% (Figure 4c). This strong linearity confirms the positive correlation between p‐n pair count and output thermopower, providing a reliable model to generate controllable Seebeck voltage. Given that ionic liquids and ionogels successfully gates OECT channels with sub‐1 V operation, Ionogel‐LT was also attempted as the gel dielectric for OECTs, which is crucial for their integration with thermoelectric devices [45, 46, 47, 48]. Electron‐transporting poly(benzimidazobenzophenanthroline) (BBL) was selected as the prototypical n‐type channel for OECTs, where the device fabrication process was illustrated in Scheme S2 and Supporting Information. Applying positive bias (V_GS_ = 0.7 V) on gate enables Li^+^ cations of Ionogel‐LT migrate through the ionogel‐semiconductor interface, exhibiting n‐type electrochemical doping of BBL and the modulation of electron carrier concentration. In comparison with BBL‐based OECT electrochemically doped by NaCl aqueous electrolyte, Ionogel‐LT also exhibits comparable doping ability with high on/off ratio owing to the excellent ionic conductivity (Figure S23a–d). To fully validate the potential on electrochemical doping capability of Ionogel‐LT, hole‐transporting semiconductor of oligo(ethylene glycol)‐functionalized polythiophene (p(g2T‐T)) was synthesized and employed as the p‐type OECT channel (Scheme S1, Figures S24 and S25). Negative bias (V_GS_ = ‐0.7 V) on gate drive TFSI^−^ anions to penetrate the bulk channel, achieving p‐type electrochemical doping (Figure S23e,f). Furthermore, to ensure Seebeck voltage generated by the thermoelectric module could be well aligned with the high transconductance (*g*_m_) regime of OECTs, *g*_m_ characteristics of OECTs based on BBL channel and P(g2T‐T) channel were systematically evaluated. Concretely, when employing Ionogel‐LT as the electrolyte, the optimal operation window of gate voltage for BBL‐based OECT and P(g2T‐T)‐based OECT lie in the range of 0.6‐0.8 V and about ‐0.2 V, respectively (Figure S23). Therefore, Ionogel‐LT demonstrated its versatile role on thermoelectric module as well as ionogel dielectric for OECTs, laying a solid foundation on the effective integration between thermoelectric modules and OECTsAfter characterizing the thermoelectric performance and electrochemical doping/dedoping capability of ionogels for *i*‐TE modules and OECTs, we integrated both components on a single substrate, and monolithic integration was achieved via inkjet printing and prepatterned fluoropolymer insulating layer [49], which defined the channel geometry by photolithography process (Figure 4d; Figures S26 and S27, Schemes S2 and S3). As illustrated in Figures 4e, 3 pairs thermoelectric ionogels including n‐type Ionogel‐LT and p‐type Ionogel‐LT‐ECl were assembled alternately to serve as the thermoelectric gate, which series connected to the source electrode. Thermoelectric gating OECTs leverages the Soret effect to generate sufficient gate voltage to drive the OECT with BBL as the channel. In concrete, when a temperature gradient (ΔT) is established across the ionogel‐based thermoelectric modules, TFSI^−^ anions in Ionogel‐LT and EMIm^+^ cations in Ionogel‐LT‐ECl directionally migrate from the hot end to the cold end via thermal diffusion, forming an ionic concentration gradient. The built‐in electric field led by the ionic concentration gradient balances ionic diffusion, thus establishing a stable Seebeck voltage upon a given ΔT. The ΔT dependent ionic Seebeck voltage can effectively gate the OECT through ionogel dielectric of Ionogel‐LT (Figure 4f Figure;S28). Obviously, the thermoelectric gating OECT exhibits high on/off ratio over 10^3^ upon relatively low temperature gradient of 20 K, comparable to that of conventional top‐gate bottom‐contact OECTs (grey dashed line in Figure 4f). To further investigate the influence of ΔT on the charge carrier transport of the thermoelectric gating OECT, output characteristic curves were systematically measured under different ΔT conditions (Figure 4g). Surprisingly, the adjustable range of drain current in thermoelectric gating OECTs largely exceeds that of thermoelectric electrolyte gated transistors with three orders of magnitude [16]. The results demonstrate that as ΔT increases from 0 to 30 K, the drain current I_DS_ in the linear regime (V_DS_ = 0.2 V) rises significantly from 2.78 µA to 170.59 µA, corresponding to a relative modulation amplitude of 6036.33%. In the saturation regime (V_DS_ = 0.6 V), I_DS_ increases from 2.72 µA to 428.8 µA, with an impressive relative enhancement of 15664.71%.

### 2.4 Stretchable and Printable Thermoelectric Gating OECT

**FIGURE 5:**
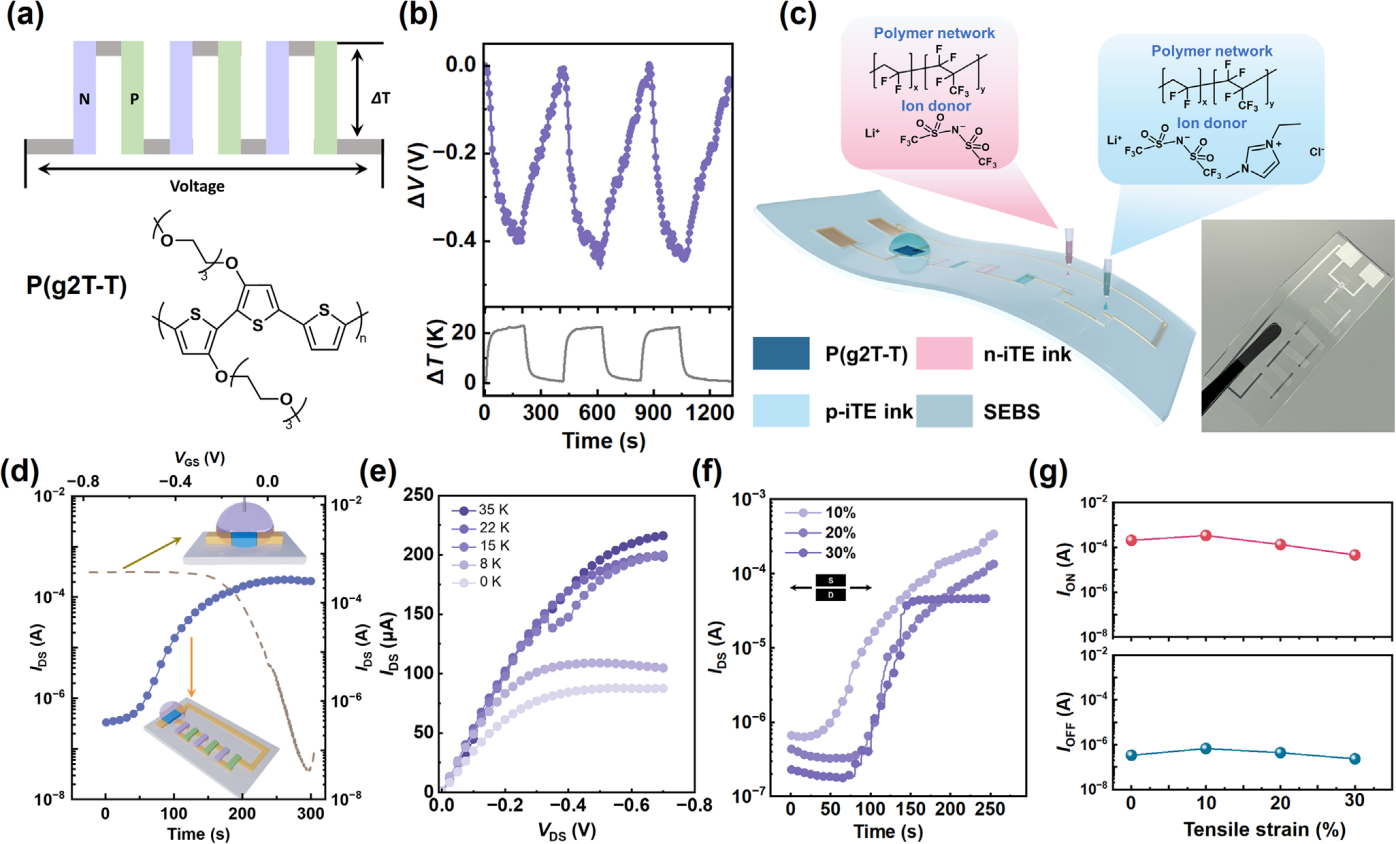
Stretchable and printable thermoelectric gating OECT. (a) Schematic illustration of redistributed *i*‐TE modules for p‐type OECT channel of p(g2T‐T). (b) Time‐dependent ΔT & ΔV curves of an *i*‐TE module with redistributed n‐p legs. (c) Schematic illustration of a typical stretchable thermoelectric gating OECT obtained by inkjet printing technique. (d) Comparative transfer curves of thermoelectric gating OECT and conventional OECT based on p(g2T‐T) channel upon applied gate voltage, and ΔT was set as 35 K. (e) Output curves at different ΔT. (f‐g) Transfer curves and I_ON_‐I_OFF_ variations of stretchable thermoelectric gating OECT upon different tensile strains.

To further verify the universality of thermoelectric gating strategy on OECTs, the distribution of the n‐leg and p‐leg in the *i*‐TE module can be rearranged to gate p‐type OECTs (Figure 5a). The reassembled *i*‐TE module generates an ionic Seebeck voltage of ‐0.4 V upon a given ΔT of 20 K, enabling the p‐type electrochemical doping of OECT (Figure 5b). We further monolithically integrated the n‐p thermoelectric module with p‐type OECT on a single substrate (Figure 5c). Here, polymer semiconductor of p(g2T‐T) was synthesized and chosen as the channel material for the p‐type accumulation‐mode OECT considering its excellent stretchability and OECT performance [50]. Furthermore, thermoplastic elastomer of styrene ethylene butylene styrene (SEBS) was employed as the flexible and transparent substrate to endow thermoelectric gating OECT with outstanding mechanical flexibility and low thermal conductivity, which is important to construct stable thermal gradient (Figures S29 and S30). To evaluate the potential influence of mechanical deformation on ion redistribution and contact resistance of ionogels, output voltages of Ionogel‐LT‐ECl upon 0% and 30% strain were compared both applied with and without ΔT. Obviously, negligible voltage change led by certain mechanical stretching was observed, indicating Seebeck voltage of thermoelectric gating OECT mainly originates from the Soret effect (Figure S31). Transfer characteristic curves of the flexible thermoelectric gating OECT with p(g2T‐T) channel was depicted in Figure 5d. Notably, upon a given ΔT of 21 K, I_DS_ exhibits pronounced thermally activated behavior within 30 s, and rapidly increases with a rate of 51.94 nA/s. The temporal change of I_DS_ obeys an exponential decay behavior, reaching dynamic equilibrium around 250 s. To gain deeper insight into the impact of ΔT on charge transport, output characteristics were systematically recorded under various ΔT conditions (Figure 5e). The results reveal that as ΔT increases from 0 to 35 K, in the linear regime (V_DS_ = ‐0.2 V), I_DS_ rises significantly from 61.29 µA to 100.66 µA, corresponding to a relative modulation amplitude of 64.24%. In the saturation regime (V_DS_ = ‐0.7 V), I_DS_ increases from 87.52 µA to 216.15 µA, with a relative enhancement of 146.97%.

As p(g2T‐T), SEBS, and ionogels are all highly stretchable, the resulting thermoelectric gating OECTs also exhibit high stretchability (Figure 5f). Transfer characteristics of the stretchable thermoelectric gating OECT upon fixed ΔT of 35 K were investigated in different uniaxial strain conditions. The thermoelectric gating OECT exhibits stable thermally activated behavior across strain ranging from 10% to 30%. Specifically, under 10% strain, the current increases at a rate of 27.63 nA/s within 30 s after applying the temperature gradient; under 20% strain, the rate decreases to 13.06 nA/s; and under 30% strain, the rate further reduces to 6.59 nA/s. The decrease in the I_DS_ rise rate with increasing strain may be attributed to the cracks formed in p(g2T‐T) film at high strain condition, impairing the efficient hole transport across the channel. However, both I_ON_ and I_OFF_ exhibit neglectable decrease in certain applied strains, indicating stable thermoelectric gating performance under stretching operation (Figure 5g).

## 3 Conclusion

This study reports a type of n‐p convertible thermoelectric ionogels, where the [EMIm][Cl] doping endows the controllable thermopower ranging from ‐3.61 mV K^−1^ (n‐type) to +9.74 mV K^−1^ (p‐type). Through spectroscopic results and MD simulations, we identified that changes in the cation‐polymer network (Li^+^‐F) and metal‐halogen (Li^+^‐Cl^−^) interactions are responsible for the transition of anion‐dominant transport to cation‐dominant transport, thus inducing the polarity reversal. Based on this, we designed an *i*‐TE module consisting of three pairs of p‐n units in series, achieving an effective Seebeck coefficient of 28.04 ± 2.56 mV K^−1^. Experimental data confirmed a linear relationship between the number of p‐n pairs and thermoelectric power (R^2^ = 99.93%). Furthermore, we integrated the *i*‐TE with a BBL‐channel organic electrochemical transistor (OECT), resulting in an integrated thermal gradient gated device with an on/off ratio exceeding 10^3^ under low thermal gradient of 20 K. We developed an integrated thermoelectric gate‐controlled OECT for flexible applications using SEBS as the substrate and p(g2T‐T) as the channel material. By reversing the arrangement of the p‐n gel pairs, we achieved a thermoelectric voltage of ‐0.4 V under 20 K, enabling the operation of a p‐type p(g2T‐T)‐based OECT. Notably, the device maintained stable electrical performance even under 30% tensile strain (with negligible degradation in *I*_ON_ and *I*_OFF_), offering an innovative solution for bioelectronics and wearable sensing applications.

## Funding

National Natural Science Foundation of China (Grant No. 22201309, 52203319, 52373315), Graduate Education Reform Project of Henan Province (Grant No. 2023SJGLX136Y), Henan Science and Technology Department Grant (252300421252), Natural Science Foundation of Hubei Province (2021CFB200).

## Conflicts of Interest

The authors declare no conflicts of interest.

## Supporting information




**Supporting File**: advs75450‐sup‐0001‐SuppMat.docx.

## Data Availability

The data that support the findings of this study are available from the corresponding author upon reasonable request.
