# Supplementary material for: Thermoelectric Gating Organic Electrochemical Transistors Enabled by Printable Ionogels With n‐p Convertible Thermopower
Source: Adv Sci (Weinh). 2026 Apr 23;13(41):e75450. doi: 10.1002/advs.75450 (PMC13335736; doi:10.1002/advs.75450)
Supplement: Supplementary file 1 — Supporting File: advs75450‐sup‐0001‐SuppMat.docx. [file ADVS-13-e75450-s001.docx]

Supporting Information

**Thermoelectric Gating Organic Electrochemical Transistors Enabled by Printable Ionogels with n-p Convertible Thermopower**

*Xingyu Hu, Xinwen Yan, Ling Huang, Xiaohang Zhang, Xuan Cao, Cong Zhang, Qingqing Sun, Hanyu Jia* and Xuying Liu**

X. Hu, L. Huang, X. Zhang, X. Cao, C. Zhang, Prof. Q. Sun, Prof. H. Jia*, Prof. X. Liu*

School of Materials Science and Engineering, Zhengzhou University, Zhengzhou 450001, China.

E-mail: jiahy@zzu.edu.cn; liuxy@zzu.edu.cn

Dr. X. Yan

Key Laboratory of Catalysis and Energy Materials Chemistry of Ministry of Education & Hubei Key Laboratory of Catalysis and Materials Science, Hubei R&D Center of Hyperbranched Polymers Synthesis and Applications, South-Central Minzu University, Wuhan 430074, China

**Table of Contents**

[**Materials and Methods** 22](#_Toc219742349)

[**Materials** 22](#_Toc219742350)

[**Synthesis of p-type semiconductor P(g2T-T)** 22](#_Toc219742351)

[**Synthesis of n- and p-type thermoelectric ionogels** 23](#_Toc219742352)

[**Device fabrication of thermoelectric gating organic electrochemical transistors.** 23](#_Toc219742353)

[**Preparation of the photosensitive fluoropolymer insulating layer** 24](#_Toc219742354)

[**Thermoelectric and electrochemical characterization** 25](#_Toc219742355)

[**Other characterizations** 26](#_Toc219742356)

[**Statistical Analysis** 26](#_Toc219742357)

[**Computational methods** 26](#_Toc219742358)

[**Supplementary Figures** 27](#_Toc219742359)

[**Supplementary References** 45](#_Toc219742360)

**Materials and Methods**

**Materials**

PVDF-HFP (molecular weight of 455, 000 g mol^-1^), ionic liquid of [EMIm][Cl] and LiTFSI, N,N-dimethylformamide were purchased from Aladdin (Shanghai, China). N-type semiconductor of BBL was obtained from westra materials company (Sweden). P-type semiconductor of P(g2T-T) was synthesized via conventional Stille cross-coupling polymerization (**Scheme S1**). The monomer of perfluoroalkylethyl acrylate (PFAEA) was purchased from Alpha (Zhengzhou, China), HDDA from Aladdin (Shanghai, China), and TAIC and AIBN from Macklin (Shanghai, China). All reagents were used as received without any further purification.

**Synthesis of p-type semiconductor P(g2T-T)**


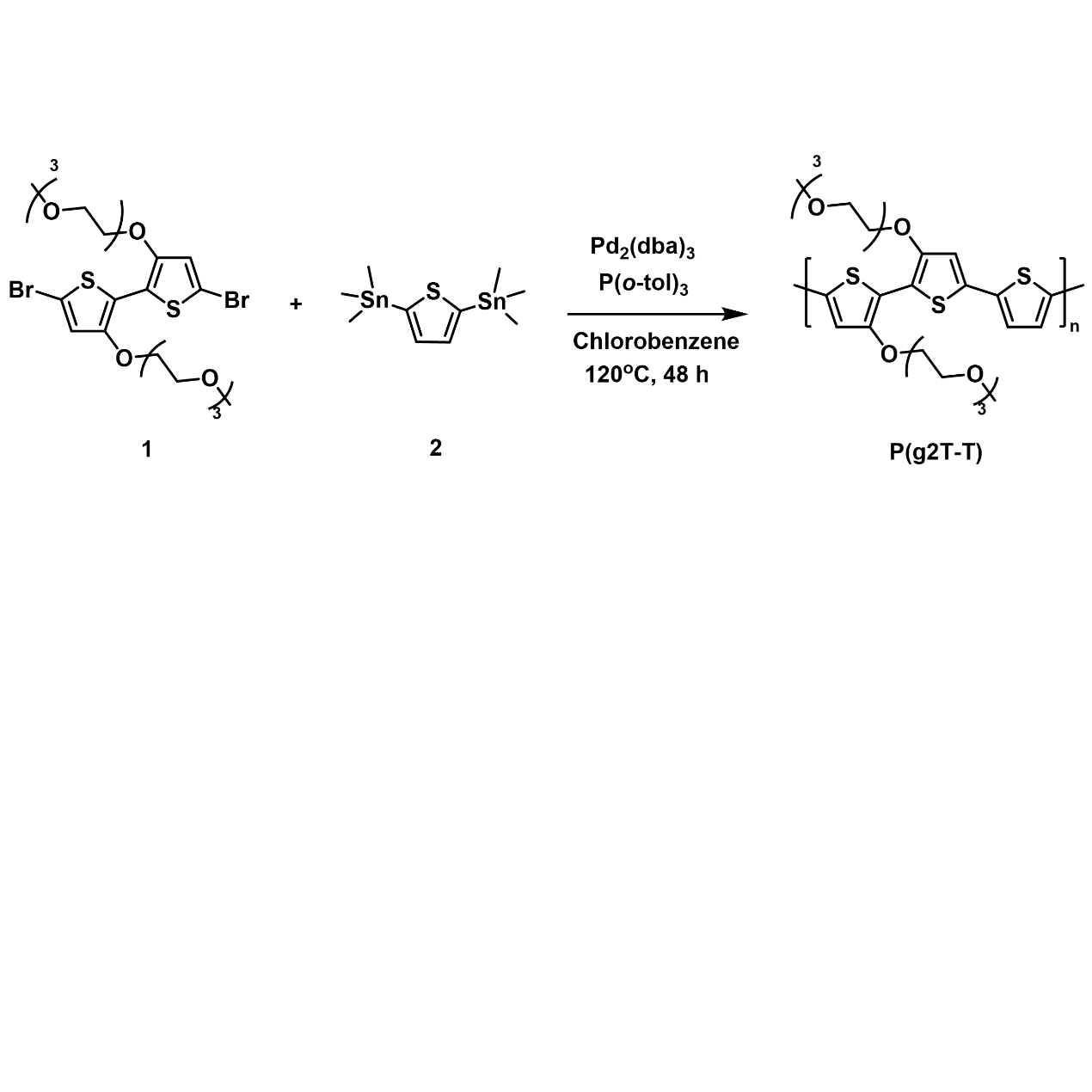


**Scheme S1**. The polymerization reaction for P(g2T-T).

The polymerization of P(g2T-T) was carried out according to Stille coupling reaction. Compound 1 of 5,5'-dibromo-3,3'-bis(2-(2-(2-methoxyethoxy)ethoxy)ethoxy)-2,2'-bithiophene was purchased from Derthon (Shenzhen, China), and compound 2 of 2,5-bis(trimethylstannyl)thiophene was purchased from Aladdin (Shanghai, China). Specifically, in a 25 mL Schlenk tube, compound 1 (194.53 mg, 0.3 mmol, 1.00 eq), compound 2 (122.93 mg, 0.3 mmol, 1.00 eq), tris(dibenzylideneacetone)dipalladium (10.99 mg, 0.012 mmol, 0.04 eq), and tri-ortho-tolyl phosphine (16.92 mg, 0.048 mmol, 0.16 eq) were added, followed by the addition of 4 mL of chlorobenzene as the solvent. Nitrogen was introduced into the Schlenk tube, and the mixture was completely frozen in liquid nitrogen, then subjected to vacuum treatment. This process was repeated three times. The mixture was heated and stirred at 120^o^C for 48 hours. After cooling to room temperature, the reaction mixture was precipitated in n-hexane to obtain the crude product. The crude product was successively extracted with n-hexane, ether, acetone, methanol, and chloroform. The product was obtained as 150.1 mg in acetone and 15.4 mg in chloroform. The product in chloroform was taken for testing and characterization.

**Synthesis of n- and p-type thermoelectric ionogels**

PVDF-HFP powder was dissolved in N,N-dimethylformamide, following with vigorous stirring at 60^o^C until transparent dispersion was obtained. Ionic liquid of LiTFSI and [EMIm][Cl] were added into the PVDF-HFP dispersion and stirred until completely dissolved. A series of thermoelectric ionogels were obtained by casting the above solution into a custom-made mold, then ensuring the solvent evaporated slowly at 70^o^C for 7 h. The mass ratio of LiTFSI ($\frac{m_{LiTFSI}}{m_{PVDF-HFP}})$ in the Ionogel-LT (n-type thermoelectric ionogel) varies from 40% to 100%. For the Ionogel-LT-ECl (p-type thermoelectric ionogel), weight ratio of [EMIm][Cl] to LiTFSI ($\frac{m_{\left[ EMIm \right][[Cl]}}{m_{LiTFSI}}$) varies from 20% to 100%.

**Device fabrication of thermoelectric gating organic electrochemical transistors.**

Source, drain, gate electrodes, and interconnects (8 nm thick Cr and 80 nm thick Ag, patterned by mask) of organic electrochemical transistor (OECT) were thermally deposited on a pre-cleaned glass substrate. The glass substrate were cleaned before use by sonication in acetone (1 time), DI water (3 times), and isopropyl alcohol (1 time), followed by nitrogen blow drying and brief oxygen plasma cleaning. Subsequently, photo sensitive fluoropolymer solution was spin-coated (1500 rpm, 90 s) onto the glass substrate to obtain the patternable insulating layer with film thickness of 1 μm. The photo sensitive fluoropolymer layer was patterned by a predesigned photomask upon UV irradiation (365 nm), then developed in acetone to define the channel and thermoelectric module areas. BBL solution (1 mg/mL) was drop-coated in the channel area at 80^o^C. Ionogel-LT is employed as the OECT electrolyte and n-type thermoelectric leg via inkjet printing. The thermoelectric module was fabricated by inkjet printing n-type leg of Ionogel-LT and p-type leg of Ionogel-LT-ECl alternately, following with annealing at 60^o^C for 1 h (**Scheme S2**).


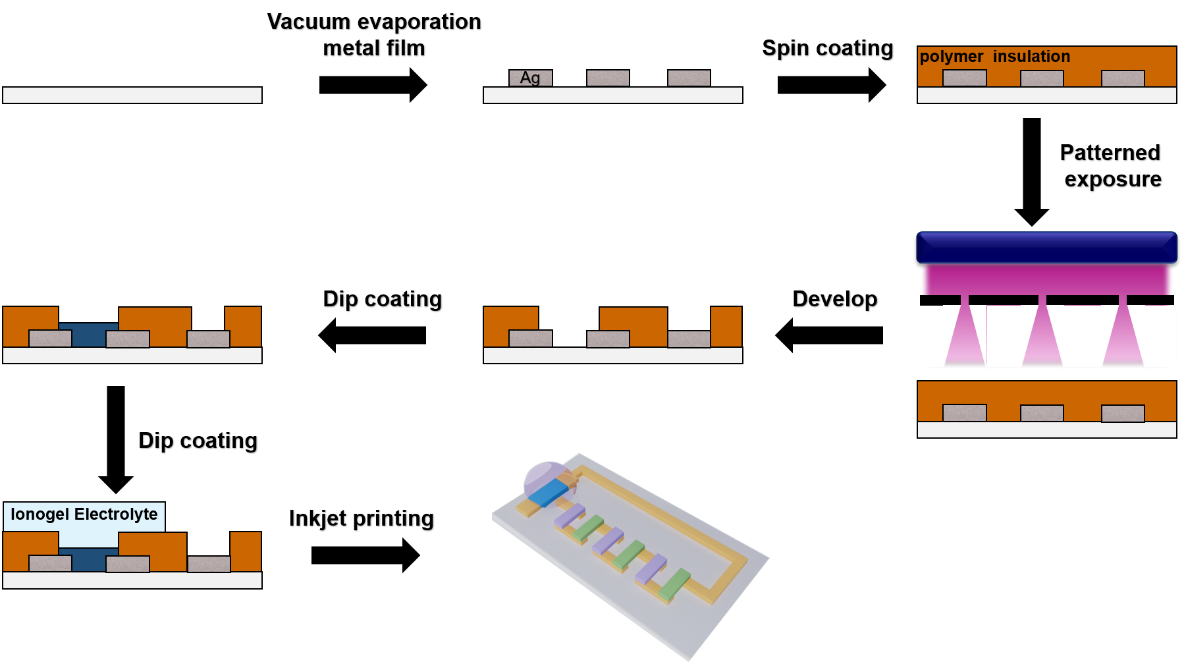


**Scheme S2**. Fabrication flow of thermoelectric gating organic electrochemical transistors.

**Preparation of the photosensitive fluoropolymer insulating layer**

The monomer of perfluoroalkylethyl acrylate (PFAEA) and the cross-linker of 1,6-hexanediyl bisacrylate (HDDA) were mixed in a mass ratio of 6:4. Subsequently, 3-5 wt% of 2,2'-azobis(2-methylpropionitrile) (AIBN) was added as the thermal initiator in the mixture with tetrahydrofuran serving as the solvent. Then, pre-polymerization was achieved by heating in a water bath at 60°C for several hours. After the pre-polymerization was completed, triallyl isocyanurate (TAIC) and the photo initiators of benzophenone and 2,2-dimethoxy-2-phenylacetophenone, was added into the mixture (**Scheme S3**).


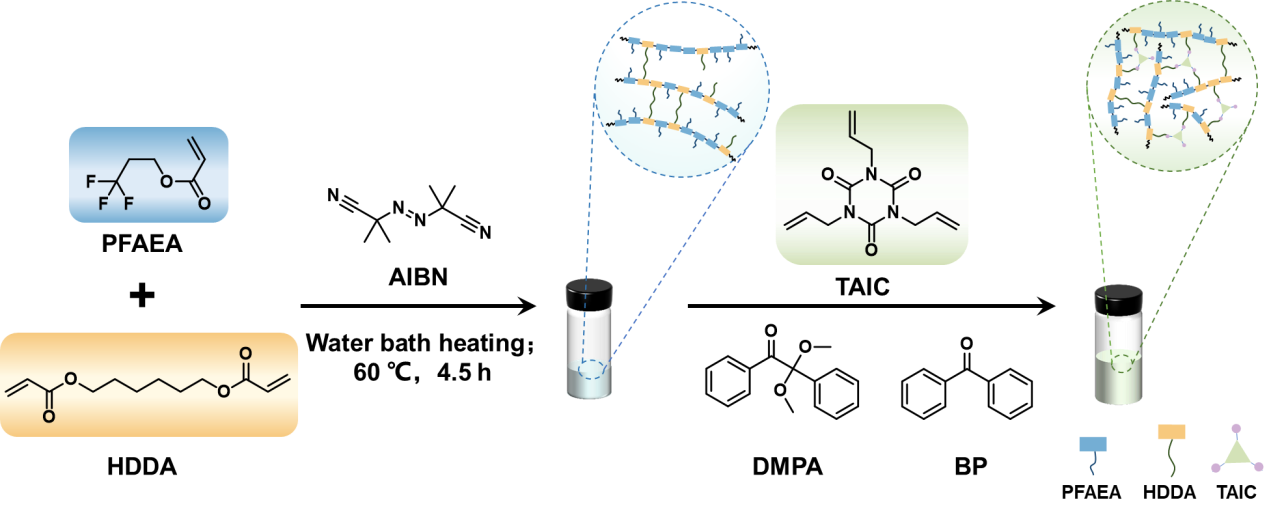


**Scheme S3**. Synthesis route of the photosensitive fluoropolymer for the patternable OECT insulating layer.

**Thermoelectric and electrochemical characterization**

Ionic Seebeck coefficients of the thermoelectric ionogels are calculated according to the equation (1),

$S_{i}=\frac{V_{H}-V_{C}}{T_{H}-T_{C}}$ (1)

where V_H_, V_C_, T_H_, and T_C_ are the voltages and temperatures at the hot end and the cold end, respectively. The open circuit voltages between the hot-cold electrodes were captured by an electrochemical workstation (DH7000C), and the applied thermal gradients were collected via the K-type thermocouples (TASI TA612C). The electrochemical workstation (DH7000C) was also used to measure the ionic resistance R_1_ (**Scheme S4**) by electrochemical impedance spectroscopy (EIS) with scan frequency ranging from 100 kHz to 0.1 Hz. The ionogels were sandwiched by two stainless steel plates to perform the EIS test. The ionic resistance R_1_ of ionogels can be obtained by fitting the EIS to an equivalent circuit (Scheme S4). The ionic conductivity (σ_i_) of the ionogels can be calculated according to the obtained R_1_, and the equation (2)

$R=\frac{1}{{}_{i}}\frac{L}{A}$ (2)

where L is the thickness, and A is the cross-sectional area of the ionogels. The energy density of thermoelectric ionogels can be calculated through the equation (3)^[1]^

$E=\frac{\int\frac{U^{2}}{R}dt}{A}$ (3)

where U, R, and A are the voltage on the external load, the external resistance, and the cross-sectional area of the ionogel, respectively. The average power density (P) on the external load was calculated by the equation (4)^[2]^,

$P=\frac{E}{\Delta t}$ (4)

where ∆t is the total duration of the stage II and IV in one thermal cycle, and the duration for stage II or IV is counted from the start to 99% of the ITEC discharge.


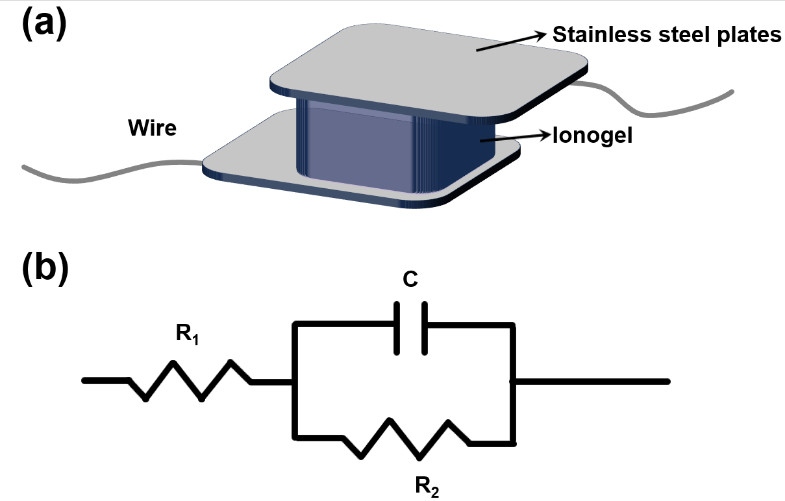


**Scheme S4**. (a) Schematic illustration for the ionic conductivity measurement of the ionogels. (b) The equivalent circuit for EIS test to calculate the ionic conductivity of the ionogels.

**Other characterizations**

The morphology change and elements mapping were performed by SEM (ZEISS GeminiSEM 360). AFM (Bruker/Nanowizard4XP) at tapping mode were employed to observe the morphology change and average roughness. The FTIR spectroscopy was acquired using Thermo Fisher iS5 with the attenuated total reflection accessory. NMR spectra were recorded via a Bruker AVANCE400. NMR spectrometer (400 MHz) in dimethyl sulfoxide. Viscosity data was measured by Discovery HR test. DSC (Differential Scanning Calorimetry) and TGA (Thermogravimetric Analysis) were obtained from Shimadzu DSC-60 Plus and Shimadzu TGA-50 test, respectively. The OECTs were characterized using a semiconductor parameter analysis tester (Keithley 4200A-SCS).

**Statistical Analysis**

All experiments were repeated independently with similar results at least three times.

**Computational methods**

**Molecular dynamics (MD) simulations**

The classical molecular dynamics (MD) simulations were carried out using GROMACS^[3]^ to understand the ionogels designed in this work. The AMBER-99 force field^[4,5]^ was used only for the Li⁺ ion, and the other molecular species (TFSI⁻ and PVDF-HFP) were parameterized using the GAFF/GAFF2 scheme with RESP charges. Specifically, RESP charges were generated at the B3LYP/6-31G* level using Multiwfn^[6,7]^, and the GAFF-based bonded and non-bonded parameters were assigned using Antechamber/acpype, which is widely adopted for ionic liquids and polymer electrolytes. No re-optimization was performed for the AMBER-99 Li⁺ parameters, as the dominant interactions in the system arise from the TFSI⁻ anion and PVDF-HFP polymer, which employ DFT-derived GAFF parameters, and our internal consistency checks (energy minimization convergence, absence of structural artifacts, and stable equilibrated trajectories) confirmed the physical reliability of the combined force-field scheme.

The linear constraint solver (LINCS) algorithm^[7,8]^ was used to constrain the bonds with hydrogen atoms. Initially, 105 LiTFSI, 41 PVDF-HFP (with x=7, y=2) were packed into a 55×55×55 Å^3^ cubic box using the packmol^[9]^ software to simulate the system 1 of LiTFSI/PVDF-HFP. Then, 207 [EMlm][Cl] molecules were added to simulate the system 2 of LiTFSI/PVDF-HFP/[EMlm][Cl]. All the systems were first heated up to target temperatures (298.15 K for RT, and 253.15 K for low T) from 10 K in 100 ps, and followed by 5 ns equilibration under isothermal-isobaric ensemble (NPT) at 1 bar. For the production run, another 15 ns NPT simulations were performed. All the MD simulations were carried out with a time step of 1 fs. For NPT simulations, the temperature was controlled by coupling the system with a Nosé-Hoover thermostat^[10]^ at time constant of 2 ps, and the pressure was controlled using the C-rescale coupling with a coupling constant of 5 ps. Electrostatic interactions were treated using the Particle-Mesh-Ewald (PME) method^[11,12]^ with a cut-off distance of 1.3 nm.

**Adsorption Energy Calculation**

All the calculations are performed in the framework of the density functional theory with the projector augmented plane-wave method, as implemented in the Vienna ab initio simulation package (vasp.6.5.1)^[13]^. The generalzied gradient approximation proposed by Perdew, Burke, and Ernzerhof is selected for the exchange-correlation potential^[14]^. The long range van der Waals interaction is described by the DFT-D3 approach^[15]^. The cut-off energy for plane wave is set to 520 eV. The energy criterion is set to 10−6 eV in iterative solution of the Kohn-Sham equation. A vacuum layer of 25 Å is added perpendicular to the sheet to avoid artificial interaction between periodic images. The Brillouin zone integration is performed using a 2x2x1 k-mesh. All the structures are relaxed until the residual forces on the atoms have declined to less than 0.02 eV/Å.

**Supplementary Figures**


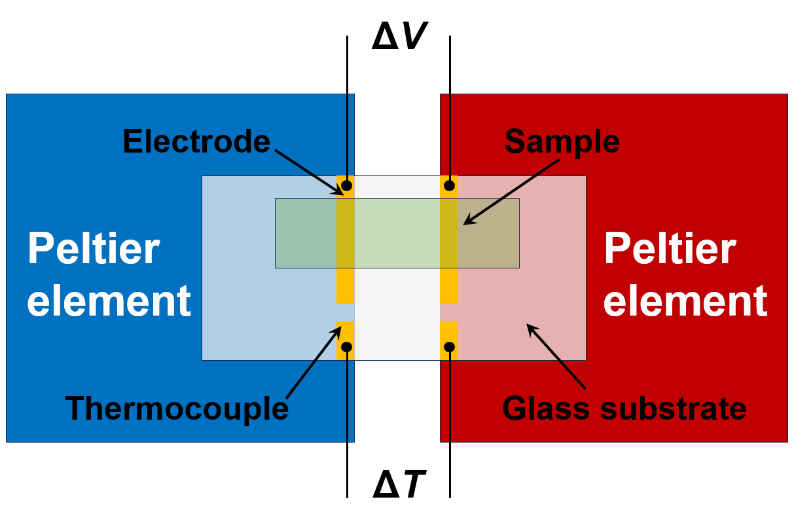


**Figure S1.** Schematic illustration of thermoelectric measurement setup for n- and p-type thermoelectric ionogels.


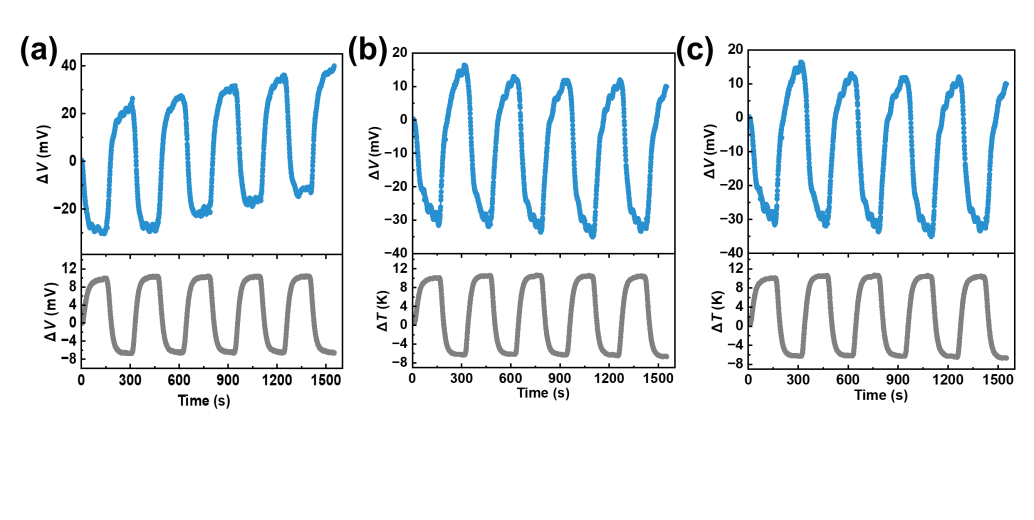


**Figure S2**. ΔV-ΔT curves of Ionogel-LT with different LiTFSI loading weights. (a) 40 wt%, (b) 60 wt%, (c)80 wt% LiTFSI.


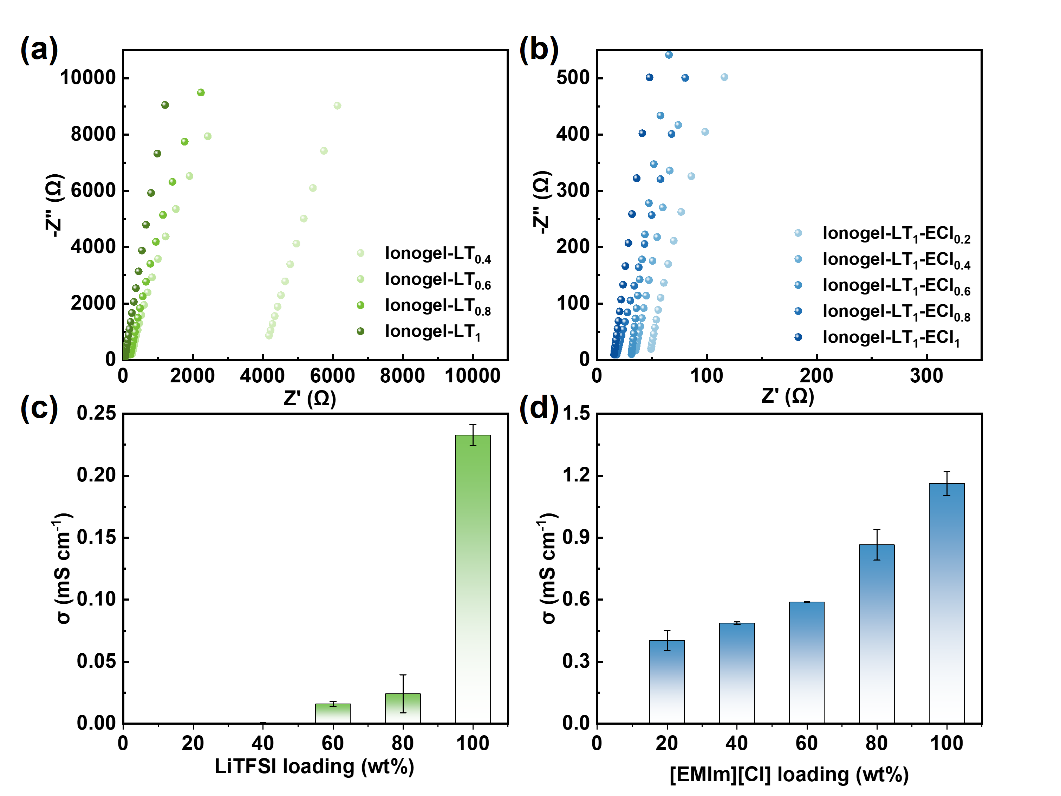


**Figure S3**. Electrochemical impedance spectra of (a) n-type thermoelectric ionogels, (b) p-type thermoelectric ionogels, and the extracted ionic conductivities of (c) n-type thermoelectric ionogels, (d) p-type thermoelectric ionogels upon different loading weights of the ionic liquid.


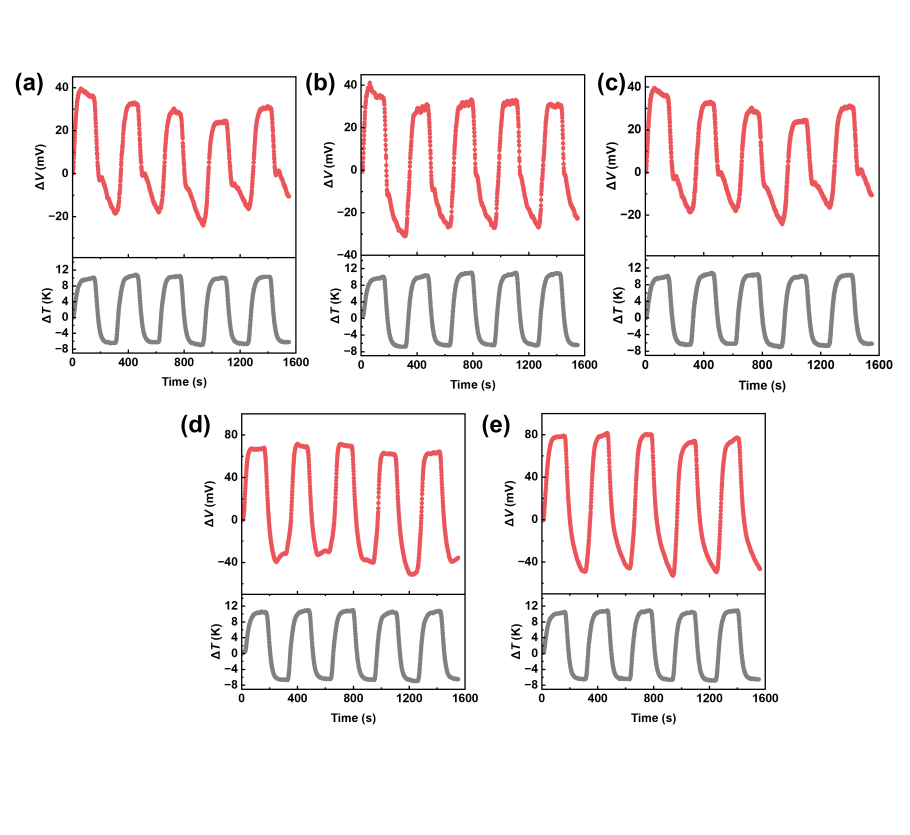


**Figure S4.** ΔV-ΔT curves for Ionogel-LT-ECl with fixed LiTFSI loading weight of 80 wt% and varied [EMIm][Cl] loading weights of (a) 20 wt%, (b) 40 wt%, (c)60 wt%, (d) 80 wt%, (e) 100 wt%.


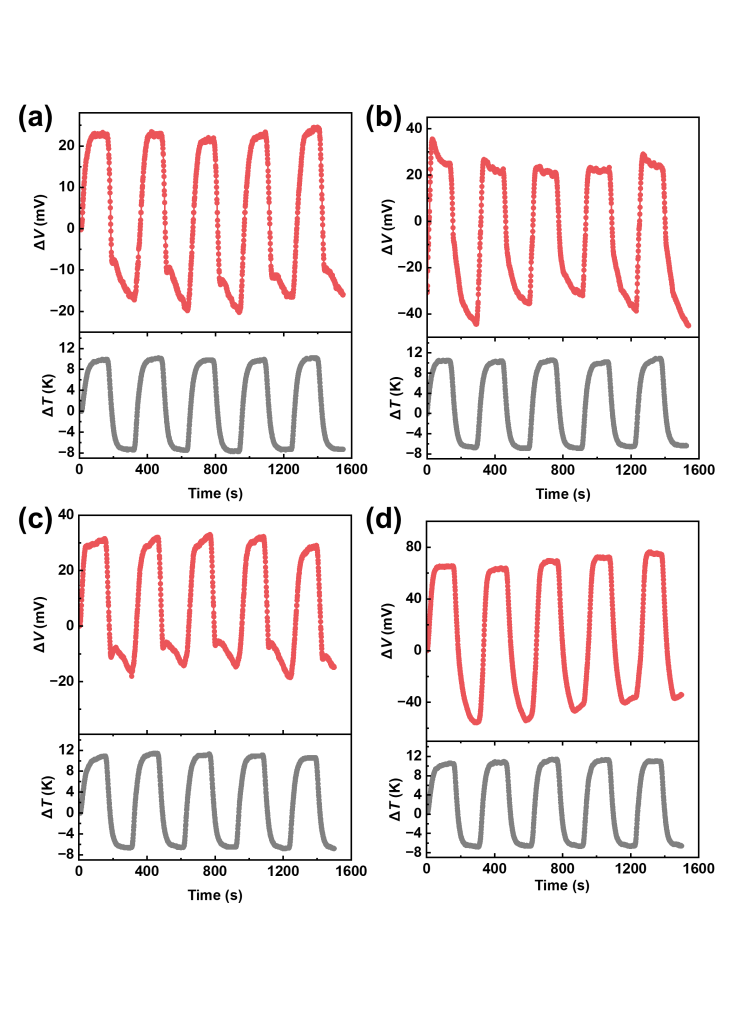


**Figure S5**. ΔV-ΔT curves for Ionogel-LT-ECl with fixed LiTFSI loading weight of 100 wt% and varied [EMIm][Cl] loading weights of (a) 20 wt%, (b) 40 wt%, (c)60 wt%, (d) 80 wt%.


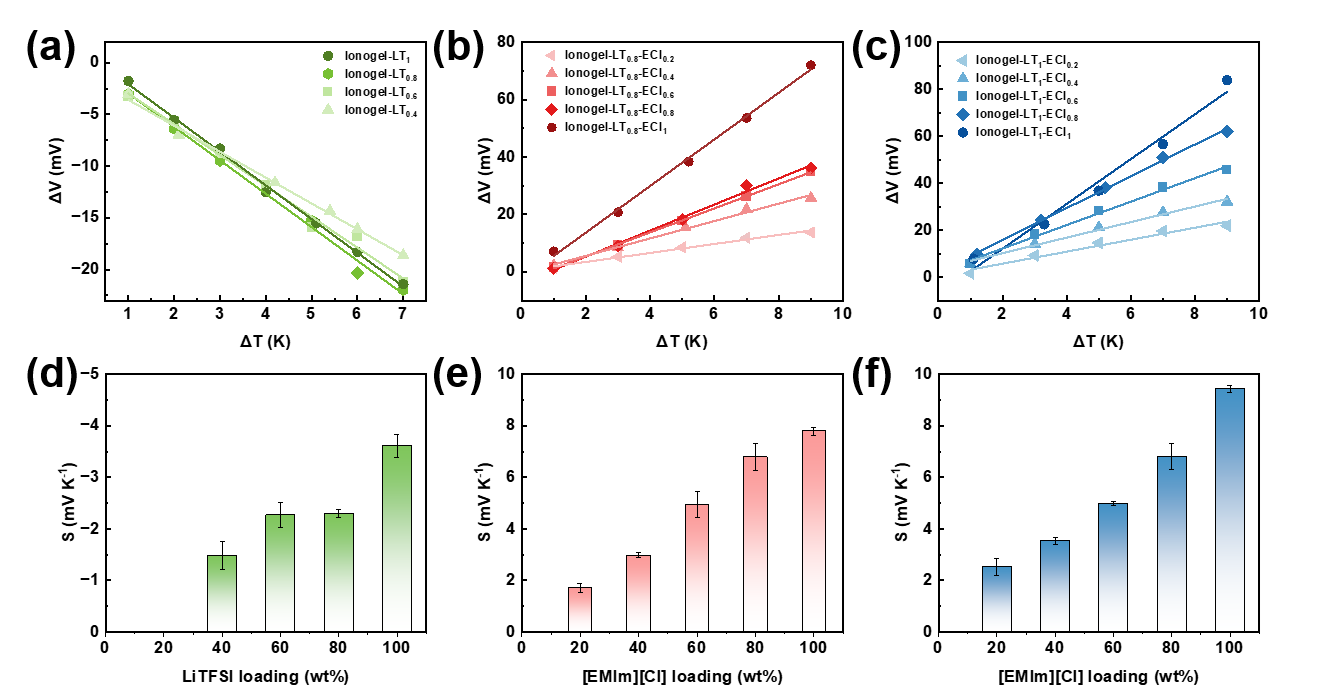


**Figure S6**. Thermoelectric performance comparison of n- and p-type thermoelectric ionogels. Measured Seebeck voltages as a function of applied temperature differences for (a) Ionogel-LT_x_, (b) Ionogel-LT_0.8_-ECl_x_, (c) Ionogel-LT_1_-ECl_x_. Seebeck coefficients of (d) Ionogel-LT_x_, (e) Ionogel-LT_0.8_-ECl_x_, (f) Ionogel-LT_1_-ECl_x_.upon different ionic liquid loading weights.


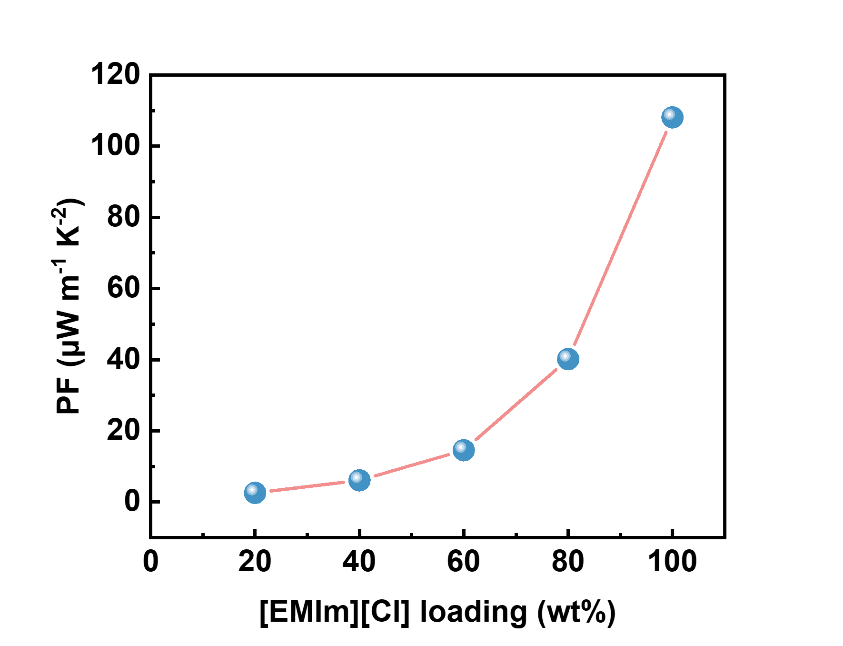


**Figure S7**. Power factors of Ionogel-LT-ECl upon different [EMIm][Cl] loading weights.


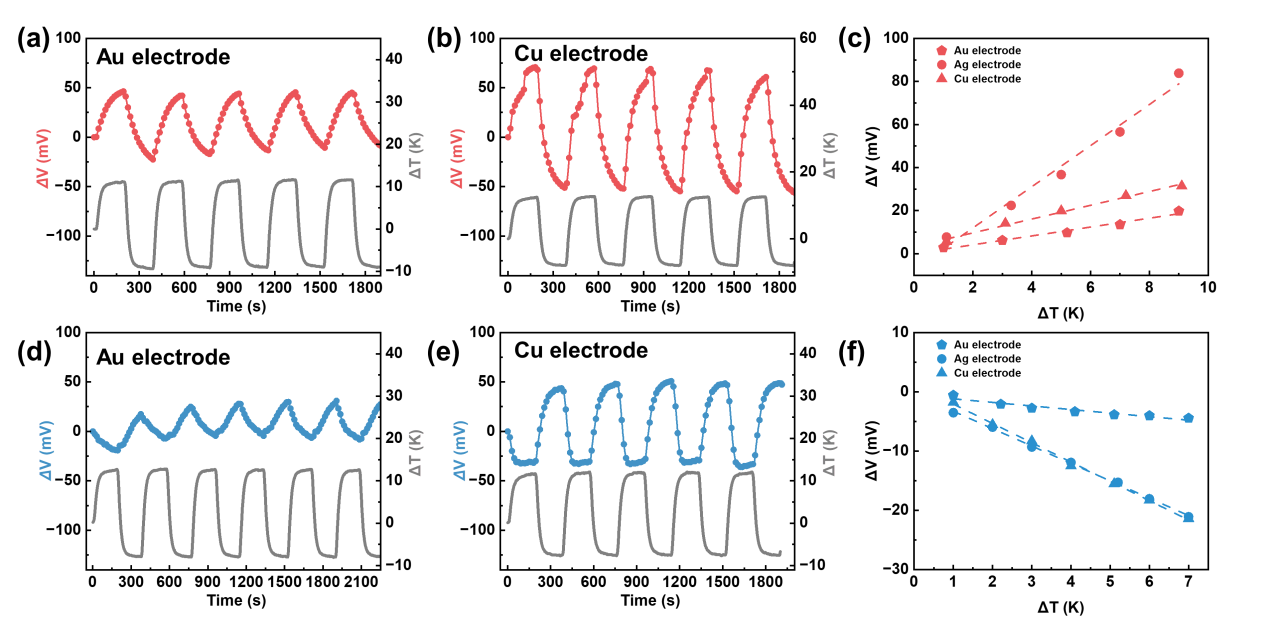


**Figure S8**. The effect of electrode materials on the thermoelectric performance of ionogels. (a-b) ΔV-ΔT curves of Ionogel-LT-ECl based on Au and Cu electrodes, respectively. (c) Seebeck coefficients of p-type Ionogel-LT-ECl upon different metal electrodes. (d), (e) ΔV-ΔT curves of Ionogel-LT based on Au and Cu electrodes, respectively. (f) Seebeck coefficients of n-type Ionogel-LT upon different metal electrodes.


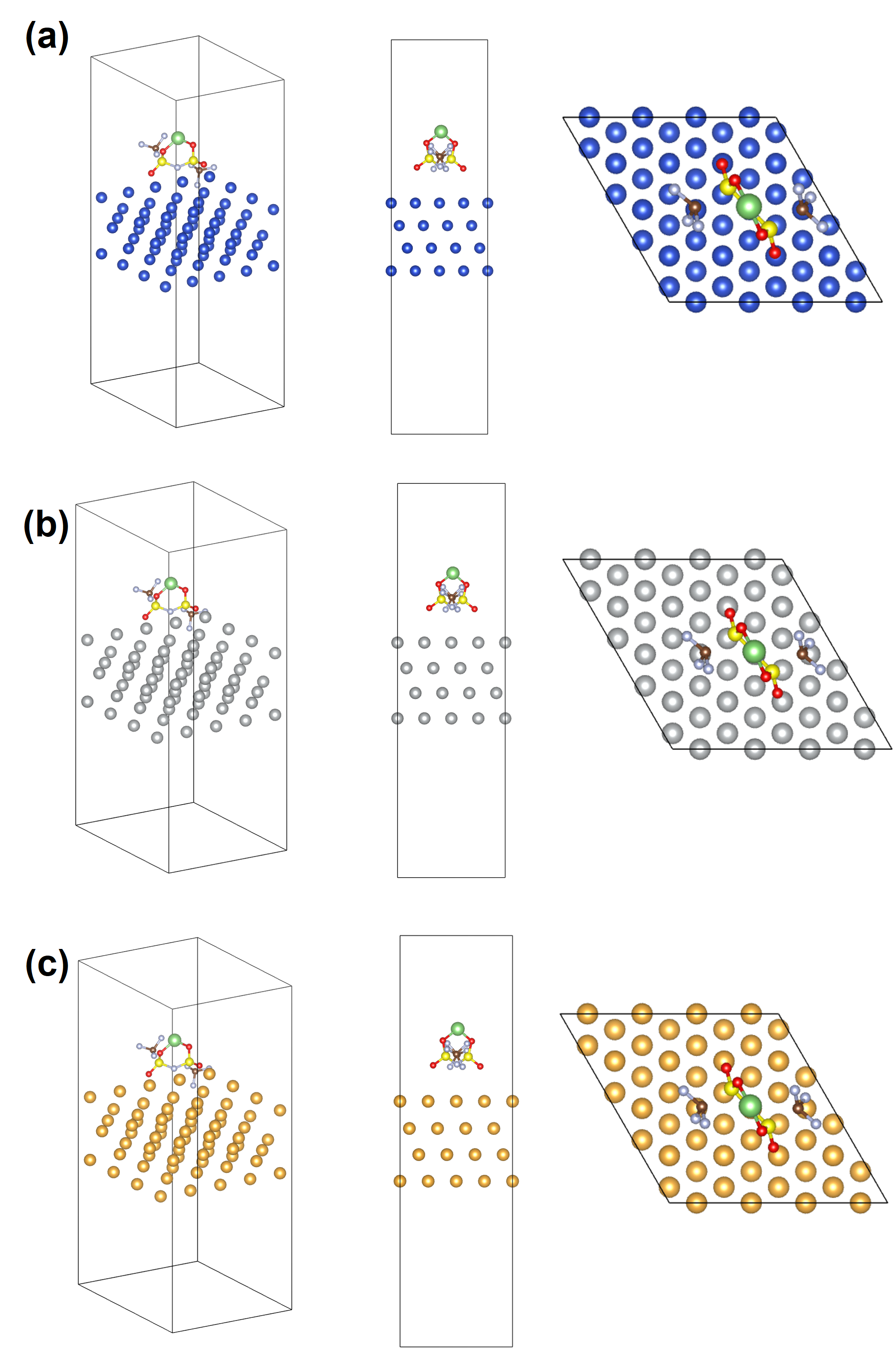


**Figure S9**. Side and top views of TFSI^-^ adsorbed on the (111) crystal planes of (a) Cu, (b) Ag, and (c) Au in the n-type Ionogel-LT.


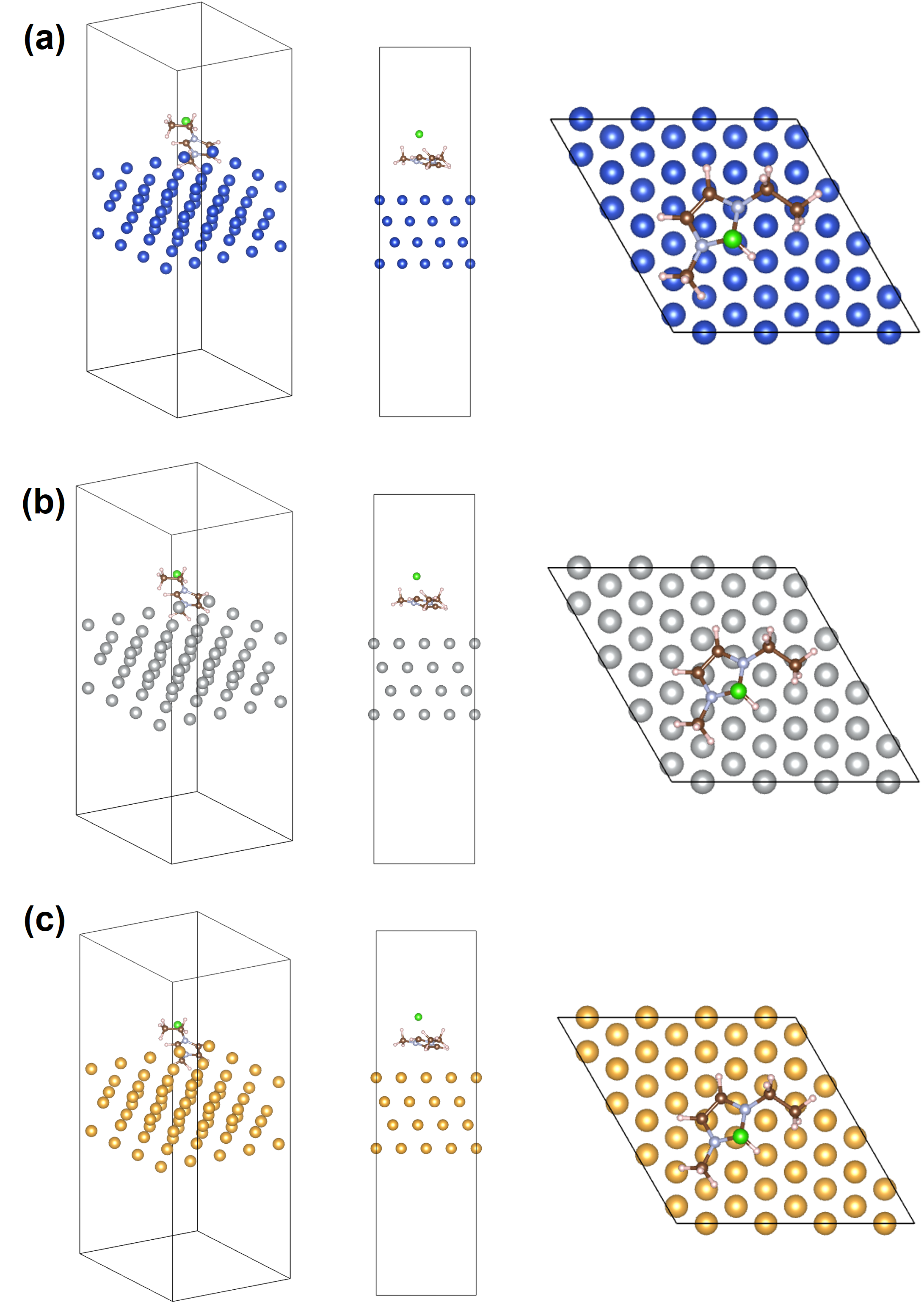


**Figure S10**. Side and top views of EMIm^+^ adsorbed on the (111) crystal planes of (a) Cu, (b) Ag, and (c) Au in the p-type Ionogel-LT-ECl.

**
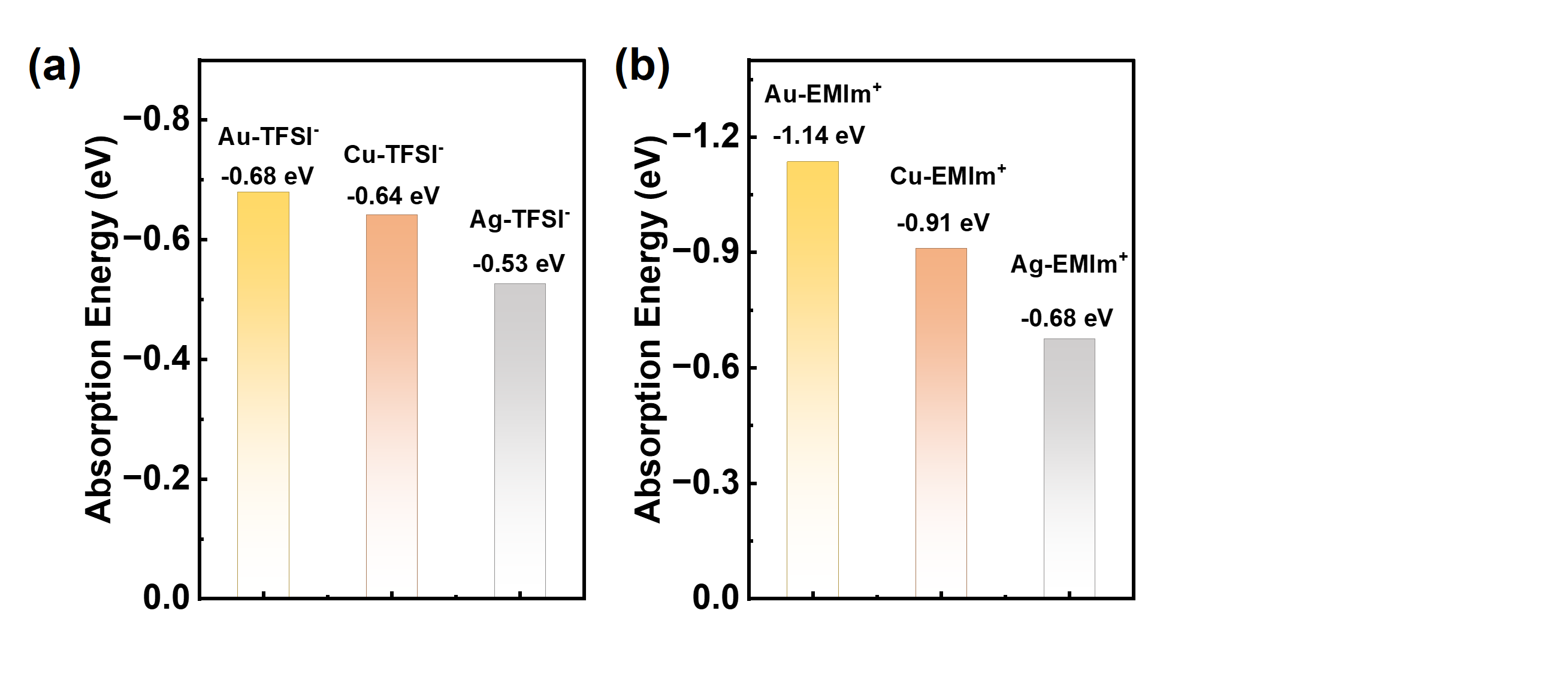
**

**Figure S11.** Adsorption energy of TFSI^-^ on Au, Ag and Cu substrate in the n-type Ionogel-LT. (b) Adsorption energy of EMIm^+^ on Au, Ag and Cu substrate in the p-type Ionogel-LT-ECl.


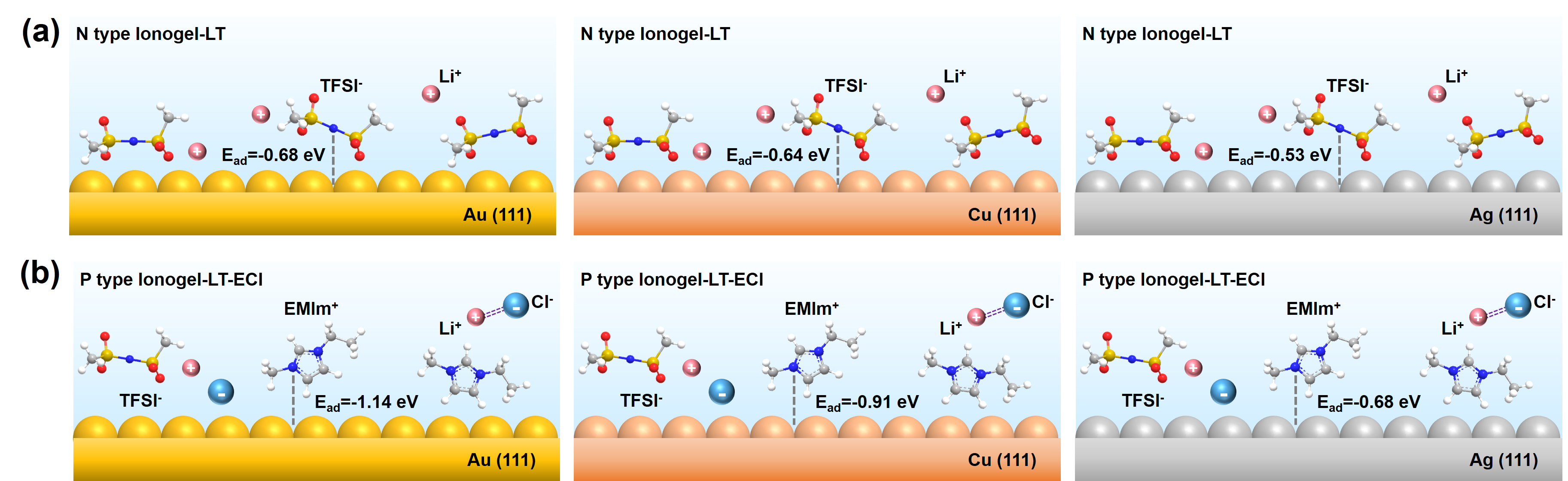


**Figure S12.** Schematic diagrams of the ion desorption behavior in (a) n type Ionogel-LT and (b) p type Ionogel-LT-ECl.


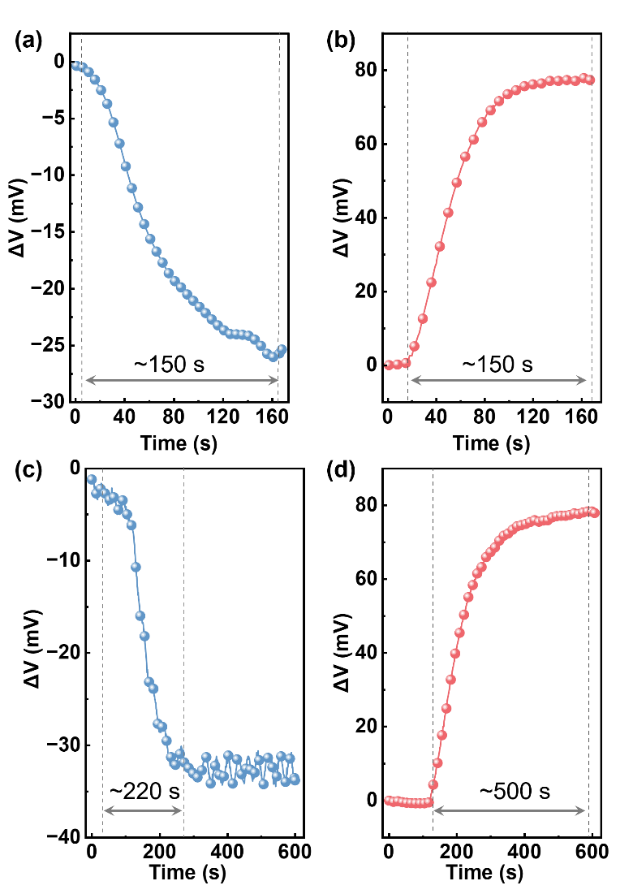


**Figure S13.** Thermal equilibration time of Ionogel-LT and Ionogel-LT-ECl based on PVDF-HFP with different HFP contents. ΔV-ΔT curves of (a) n-type Ionogel-LT and (b) p-type Ionogel-LT-ECl based on PVDF-HFP with 10% HFP content. ΔV-ΔT curves of (c) n-type Ionogel-LT and (d) p-type Ionogel-LT-ECl based on PVDF-HFP with 18% HFP content.


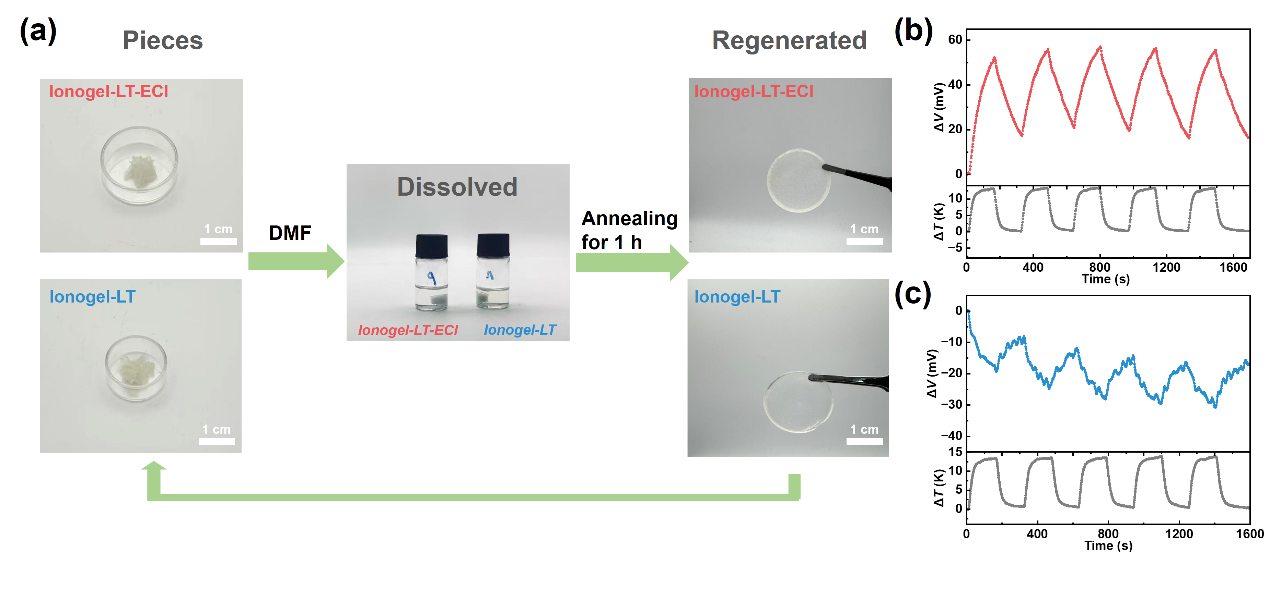


**Figure S14**. Recyclable thermoelectric ionogels. (a) Flow diagram of recyclability and reconstitution of n-type Ionogel-LT and p-type Ionogel-LT-ECl. (b) Thermoelectric properties of regenerated n-type Ionogel-LT and p-type Ionogel-LT-ECl.


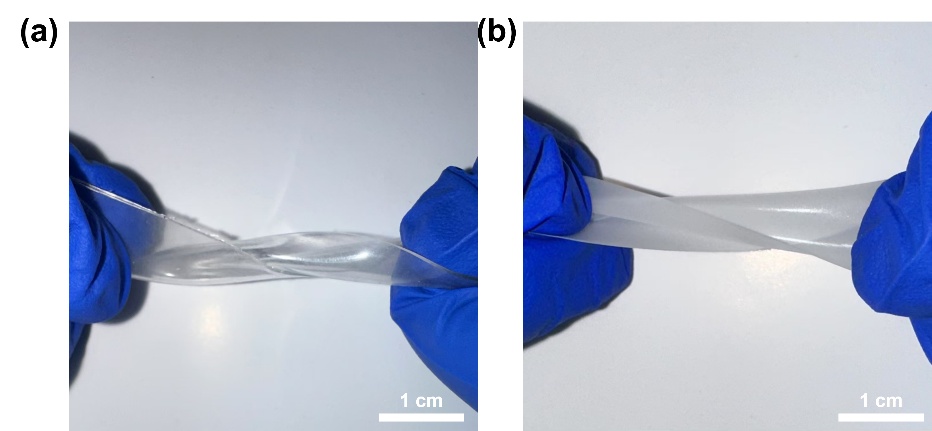


**Figure S15**. Flexibility of solid-state thermoelectric ionogels. (a) N-type Ionogel-LT, (b) p-type Ionogel-LT-ECl.


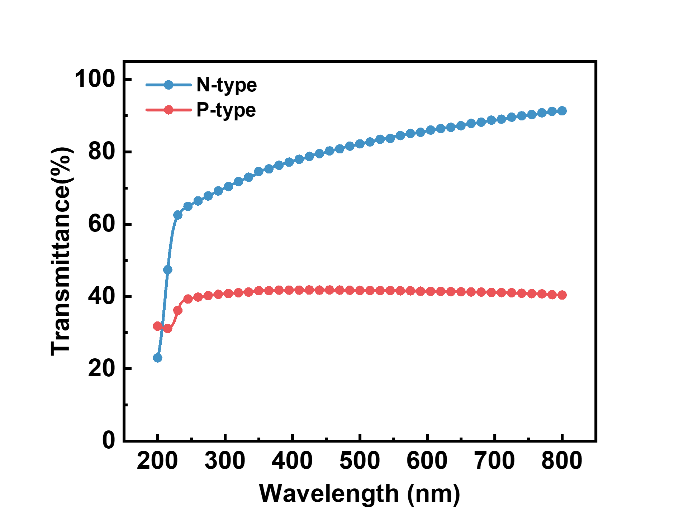


**Figure S16**. Optical properties of n-type Ionogel-LT and p-type Ionogel-LT-ECl.


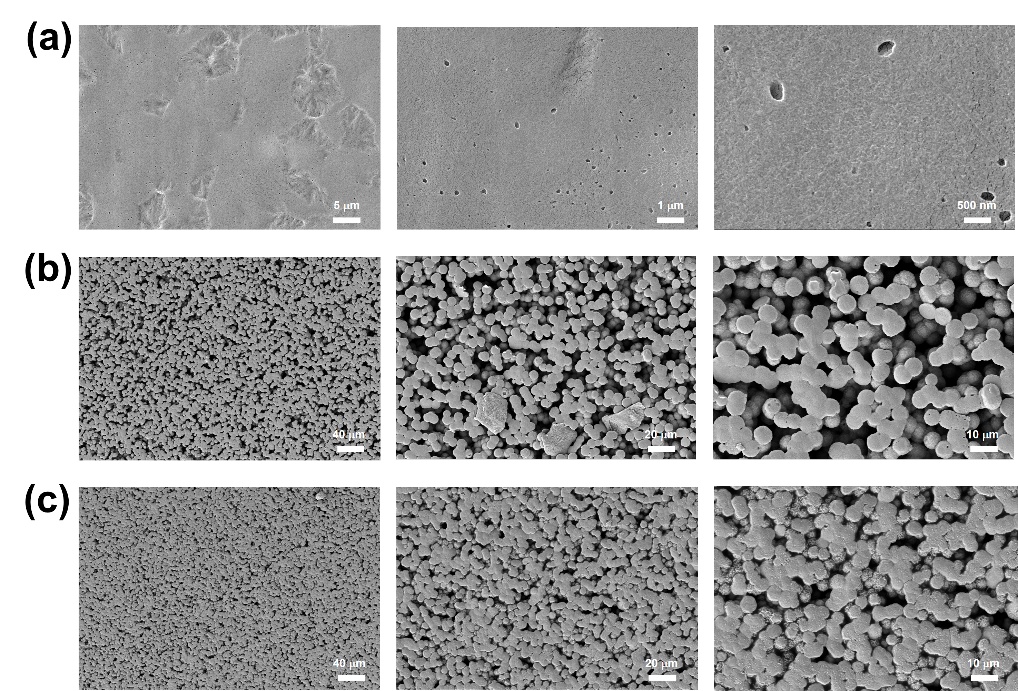


**Figure S17**. SEM images (top view) of (a) pristine PVDF-HFP, (b) n-type Ionogel-LT, and (c) p-type Ionogel-LT-ECl in different magnifications.


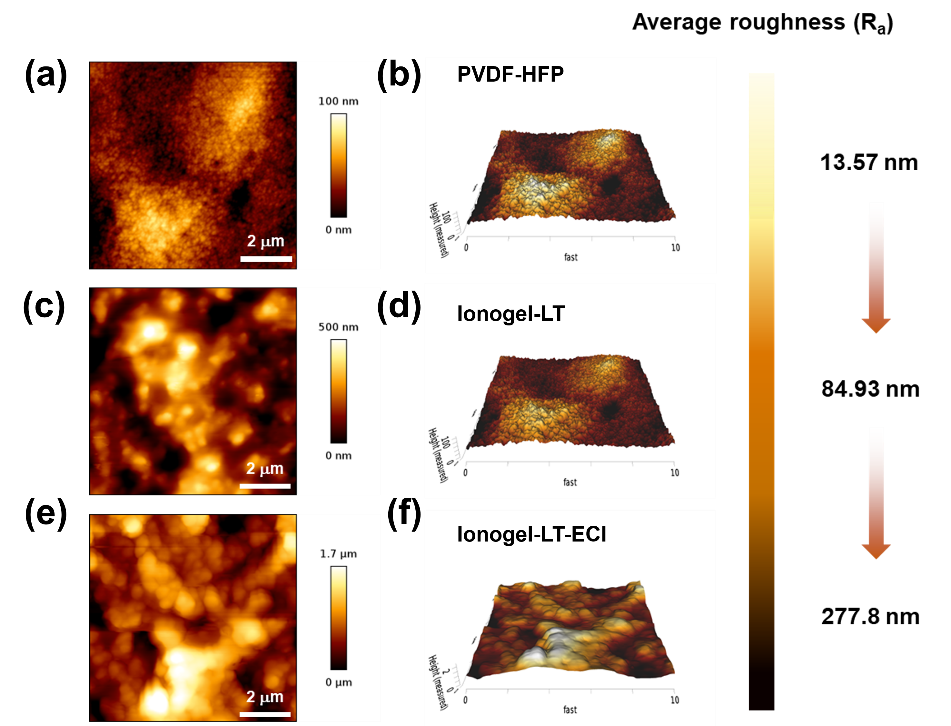


**Figure S18**. AFM images of (a) pristine PVDF-HFP, (c) Ionogel-LT, and (e) Ionogel-LT-ECl. Topographic (3D height) images of (b) pristine PVDF-HFP, (d) Ionogel-LT, and (f) Ionogel-LT-ECl.


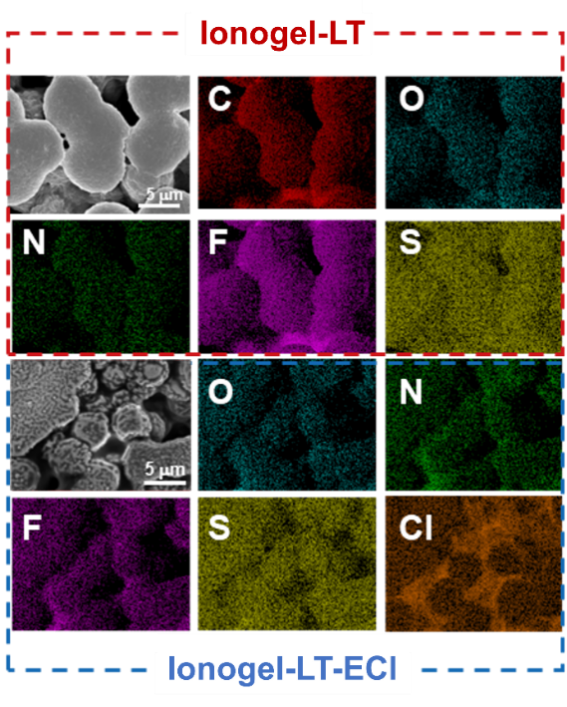


**Figure S19**. Energy-dispersive X-ray spectroscopy (EDS) mapping of element distribution in the n-type Ionogel-LT and p-type Ionogel-LT-ECl ionogels.


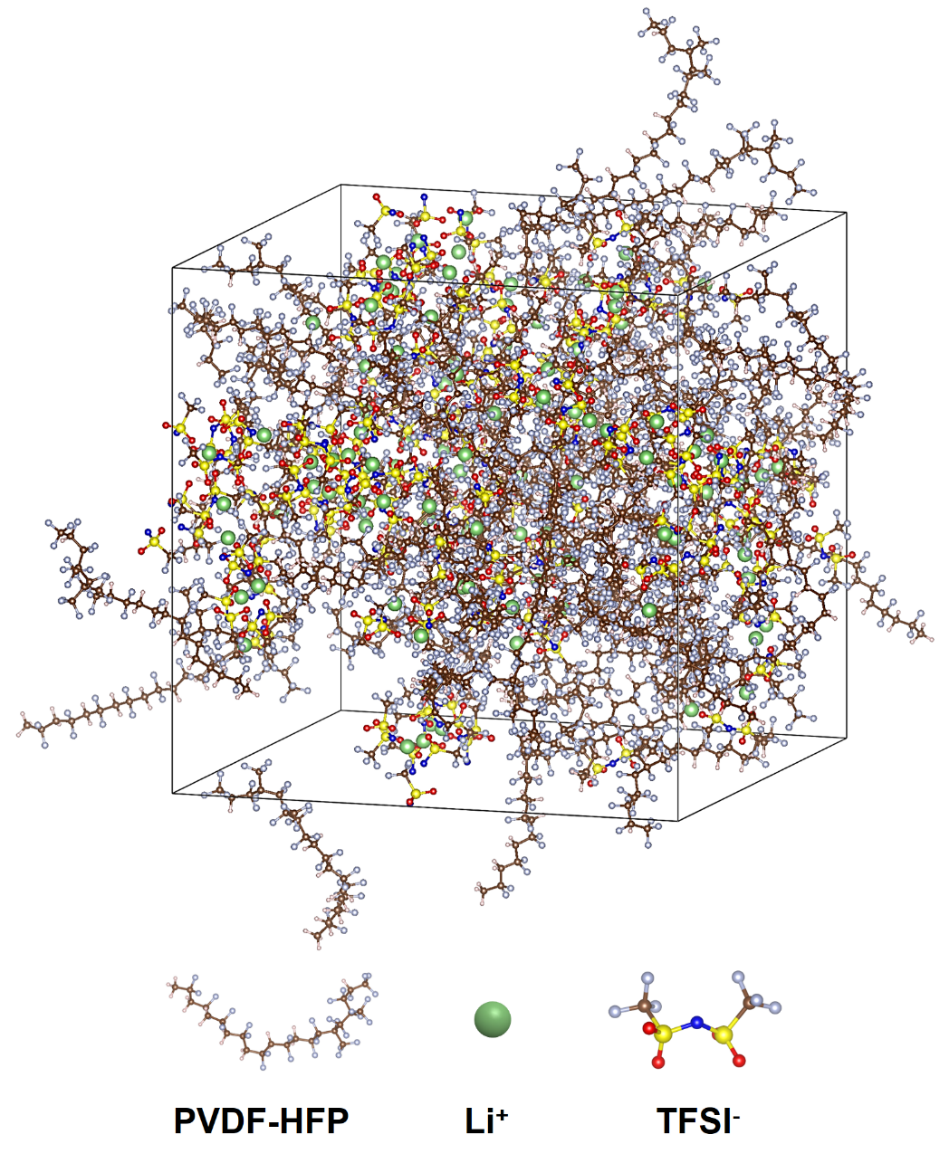


**Figure S20**. MD snapshots of Ionogel-LT. Atomic structures of PVDF-HFP and LiTFSI are shown at the bottom.


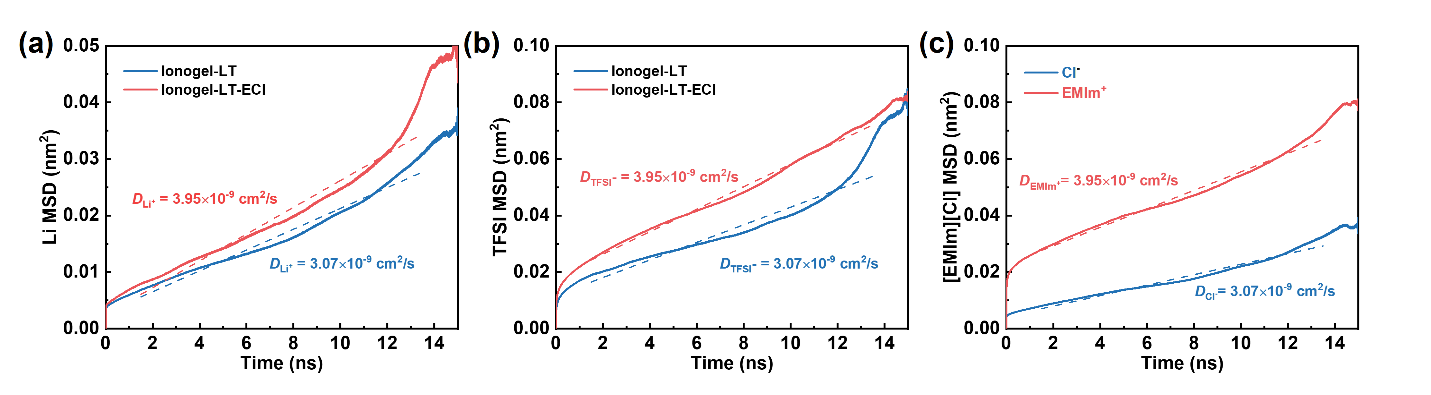


**Figure S21**. The mean-squared displacement of (a) Li^+^, (b) TFSI^-^ and (c) [EMIm][Cl].


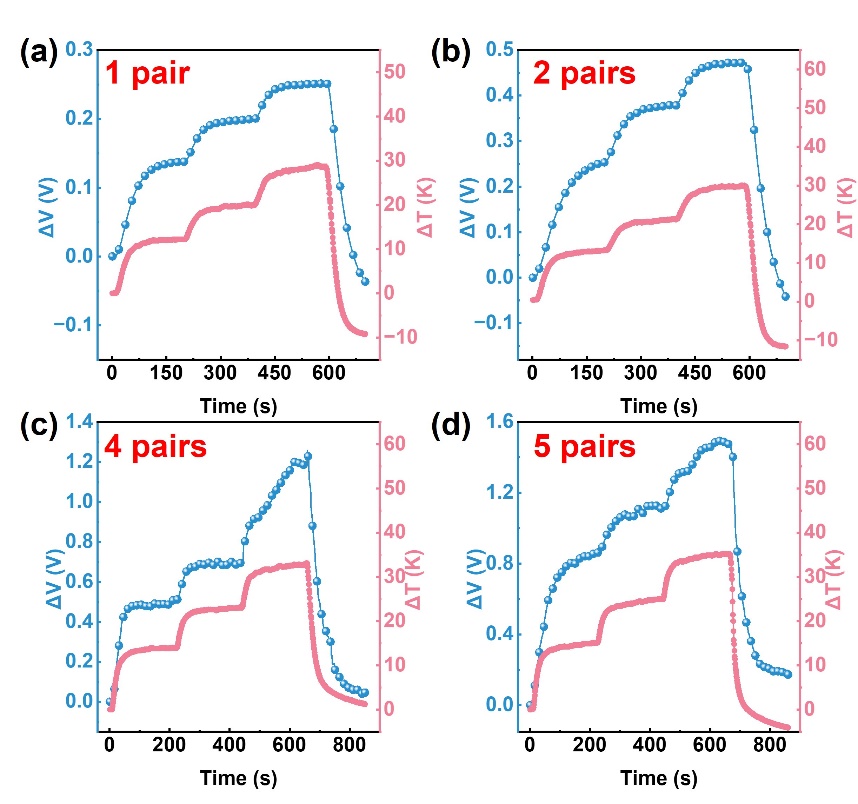


**Figure S22.** ΔV-ΔT curves for different P-N pairs of ionogel-based thermoelectric modules.


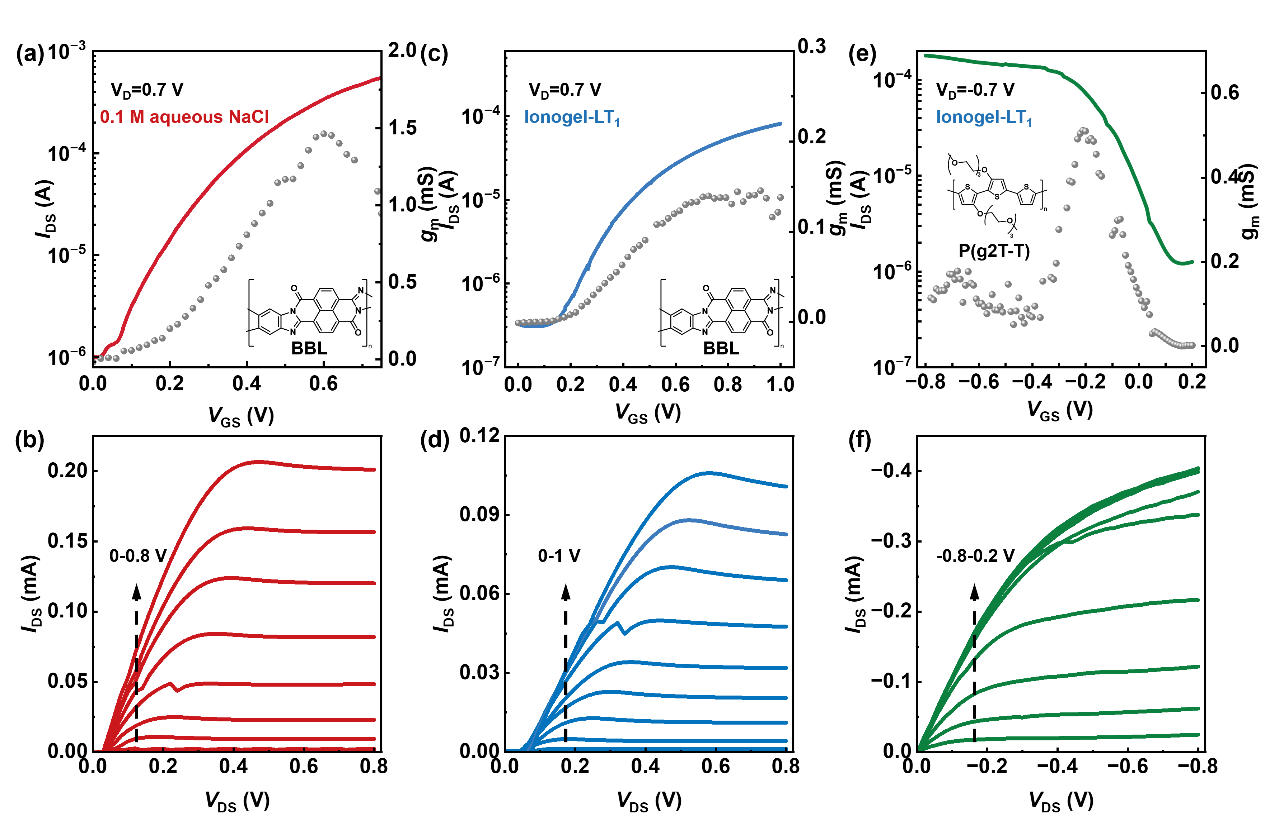


**Figure S23.** Ionogel-LT-based organic electrochemical transistors. (a-b) Transfer, transconductance, and output characteristics of n-type BBL-based OECT (W/L=500:50 μm/μm, the electrolyte is 0.1 M NaCl aqueous solution). (c-d) Transfer, transconductance, and output characteristics of n-type BBL-based OECT (W/L=500:50 μm/μm, the electrolyte is Ionogel-LT). (e-f) Transfer, transconductance and output curves of p-type P(g2T-T)-based OECT (W/L=500:50 μm/μm, the electrolyte is Ionogel-LT).


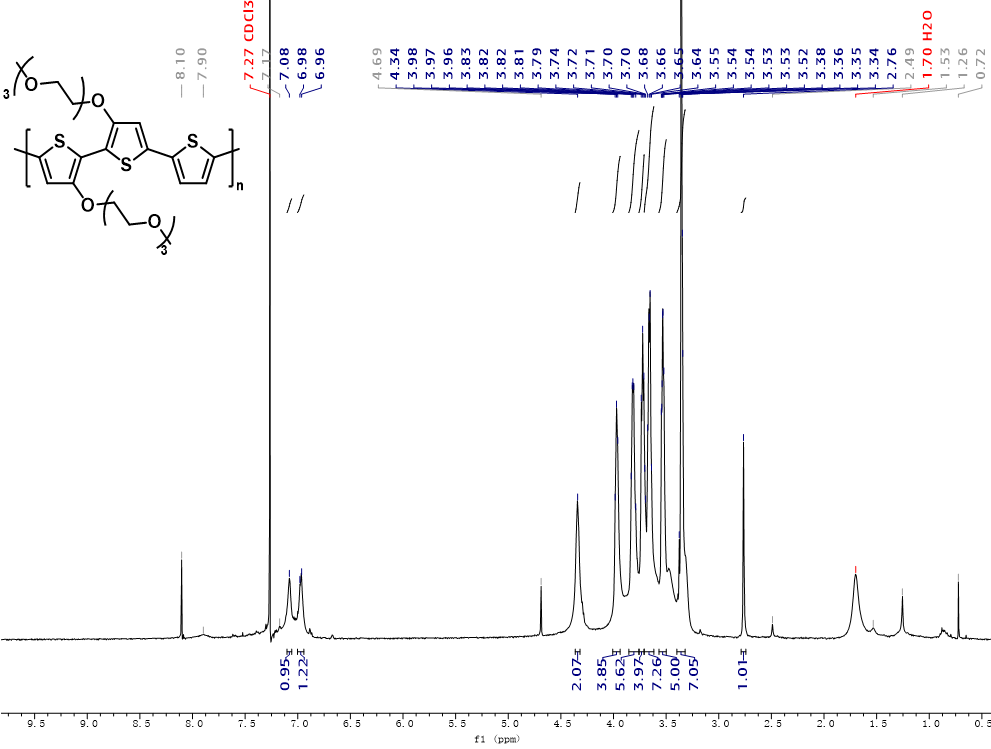


**Figure S24**. ^1^H NMR spectrum of P(g2T-T).


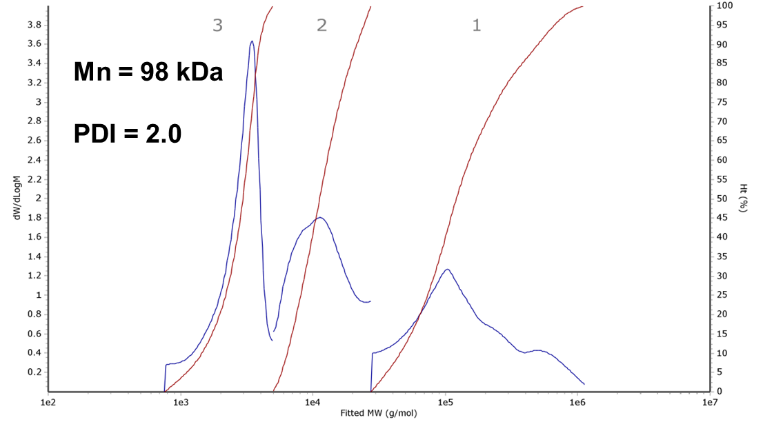


**Figure S25**. Molecular weights and polymer dispersity index (PDI) of P(g2T-T) measured in chloroform eluent.


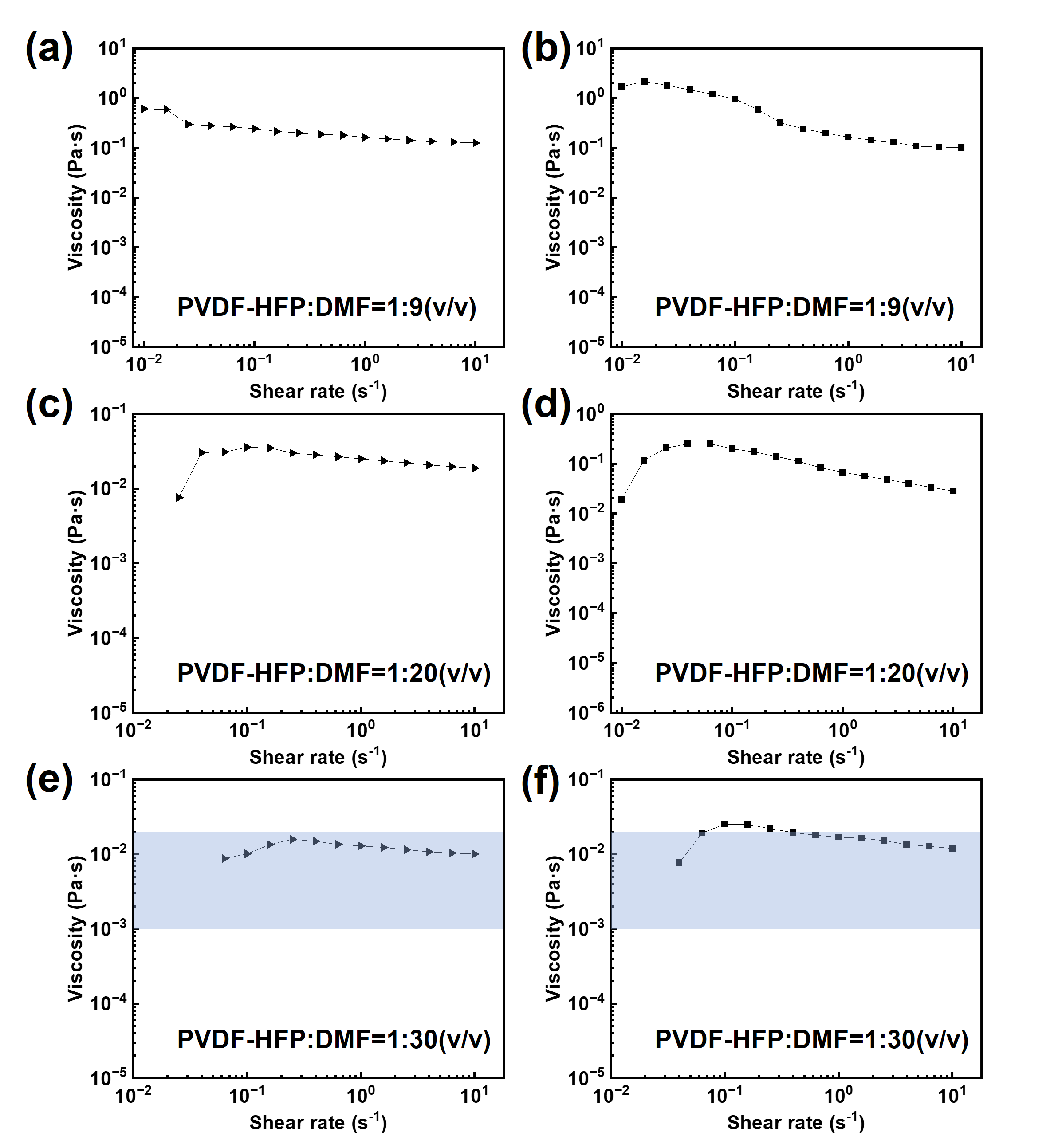


**Figure** **S26.** Rheological properties of n-type Ionogel-LT and p-type Ionogel-LT-ECl inks with DMF as solvent, the volume ratio of PVDF-HFP and DMF was controlled as 1:9 in (a-b), 1:20 in (c-d), and 1:30 in (e-f). The blue background in (e-f) highlights the suitable ink viscosity for inkjet printing technique.^[13]^


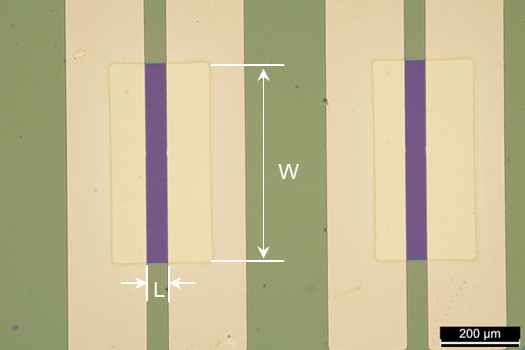


**Figure S27.** Optical images of patterned channels based on fluoropolymer insulating layers (width=500 μm, length=50 μm).


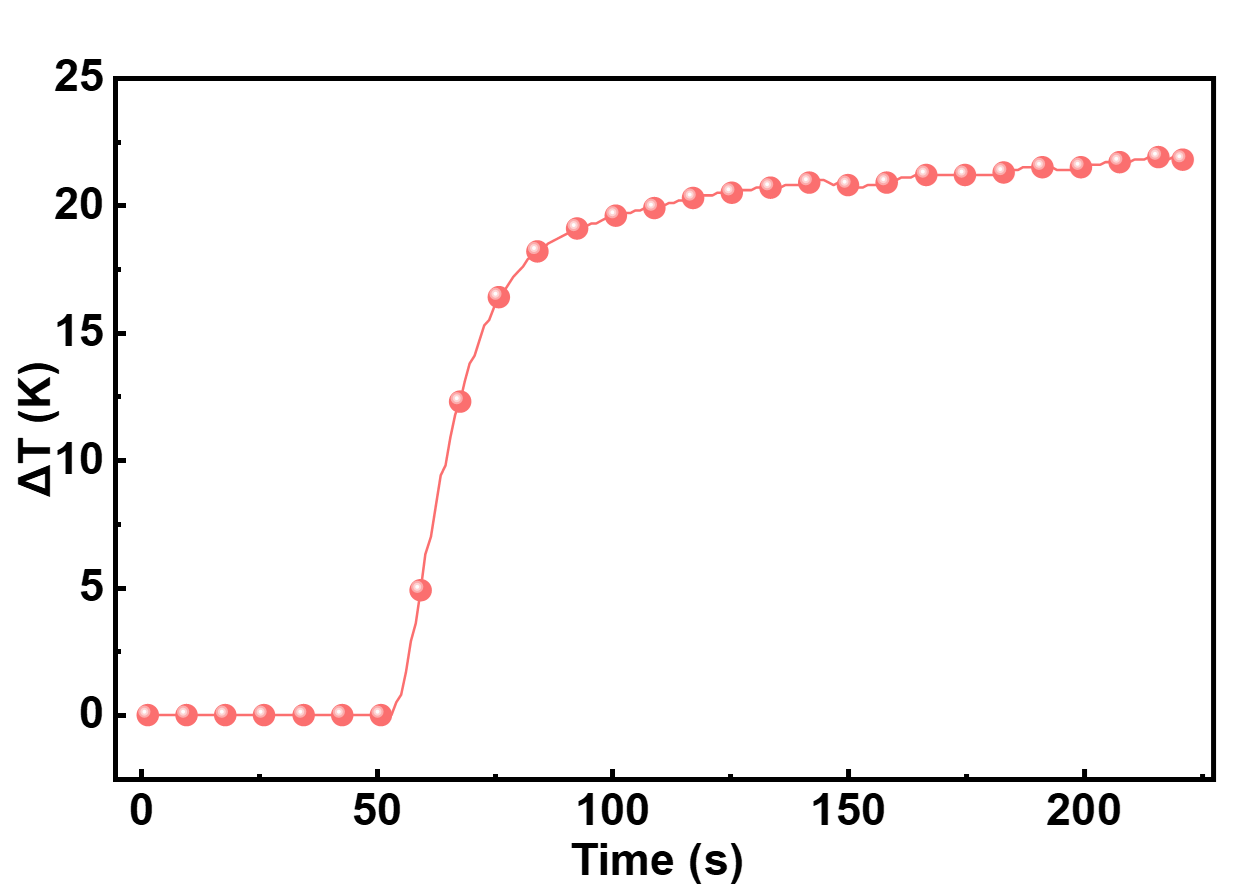


**Figure S28.** Temporal ΔT curve corresponding to Figure 4f.


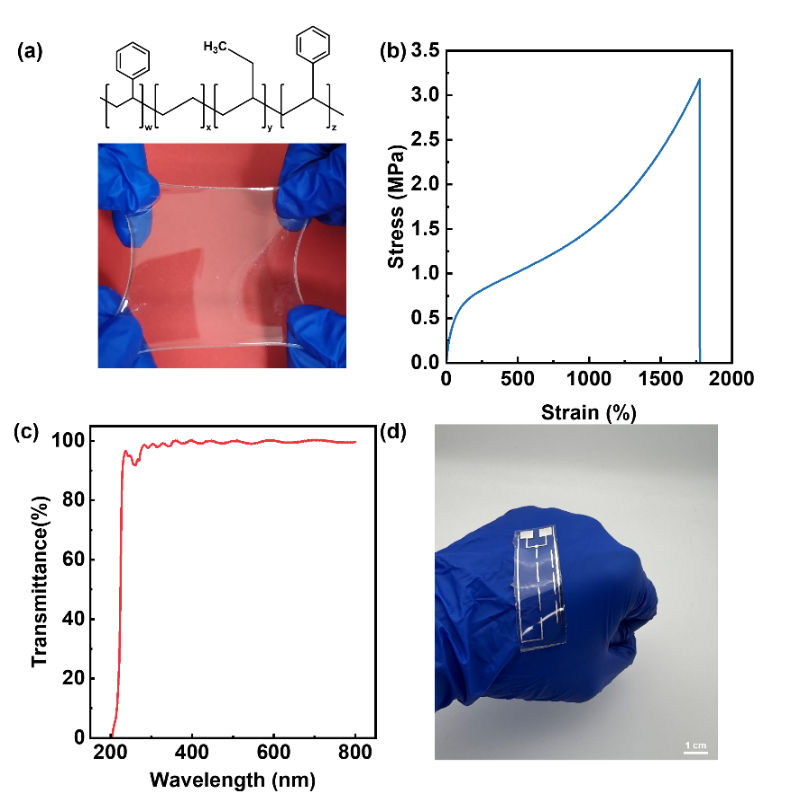


**Figure S29.** Stretchable substrate for thermoelectric gating OECTs. (a) Molecular structure and optical image of SEBS substrate. (b-c) Stress-strain curve and optical property of SEBS substrate. (d) Optical image of the all-in-one thermoelectric gating OECT.


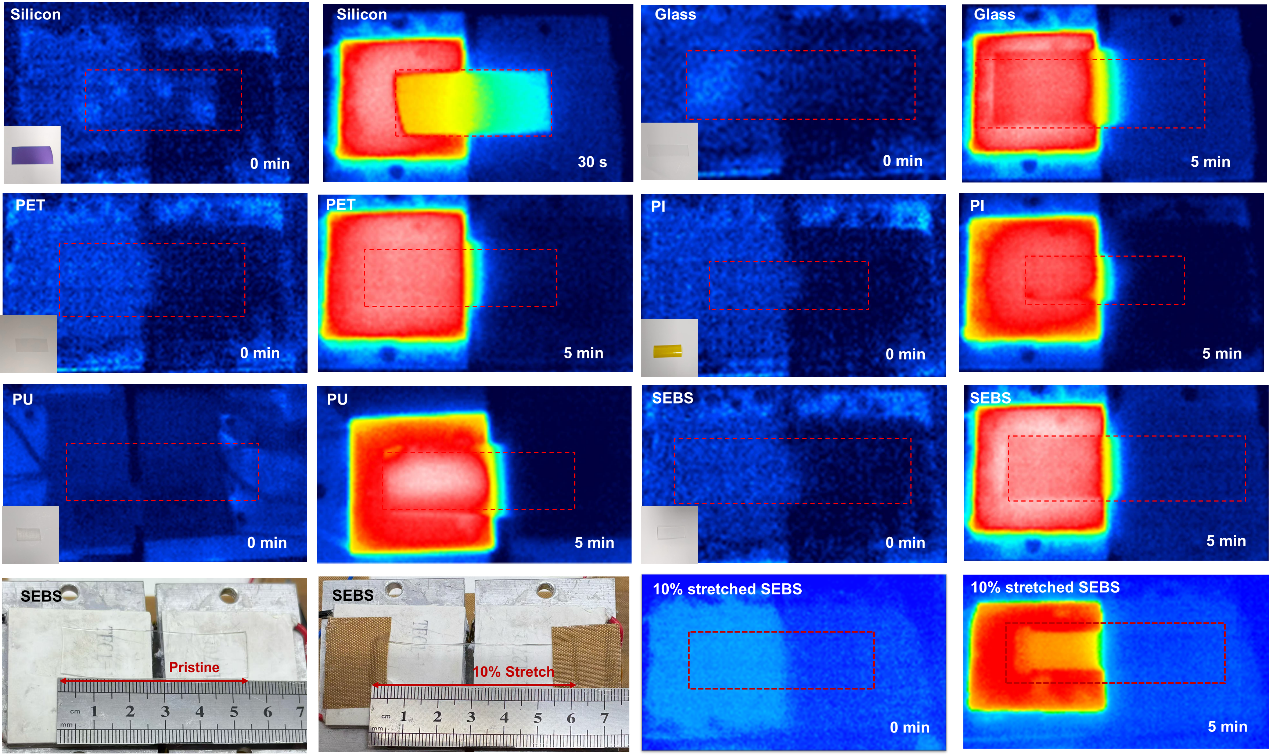


**Figure** **S30.** Heat transfer rates of different substrates for thermoelectric gating OECTs, including silicon substrate covered with oxide layer, glass substrate, and polymeric substrates of PET, PI, PU, PDMS, SEBS.


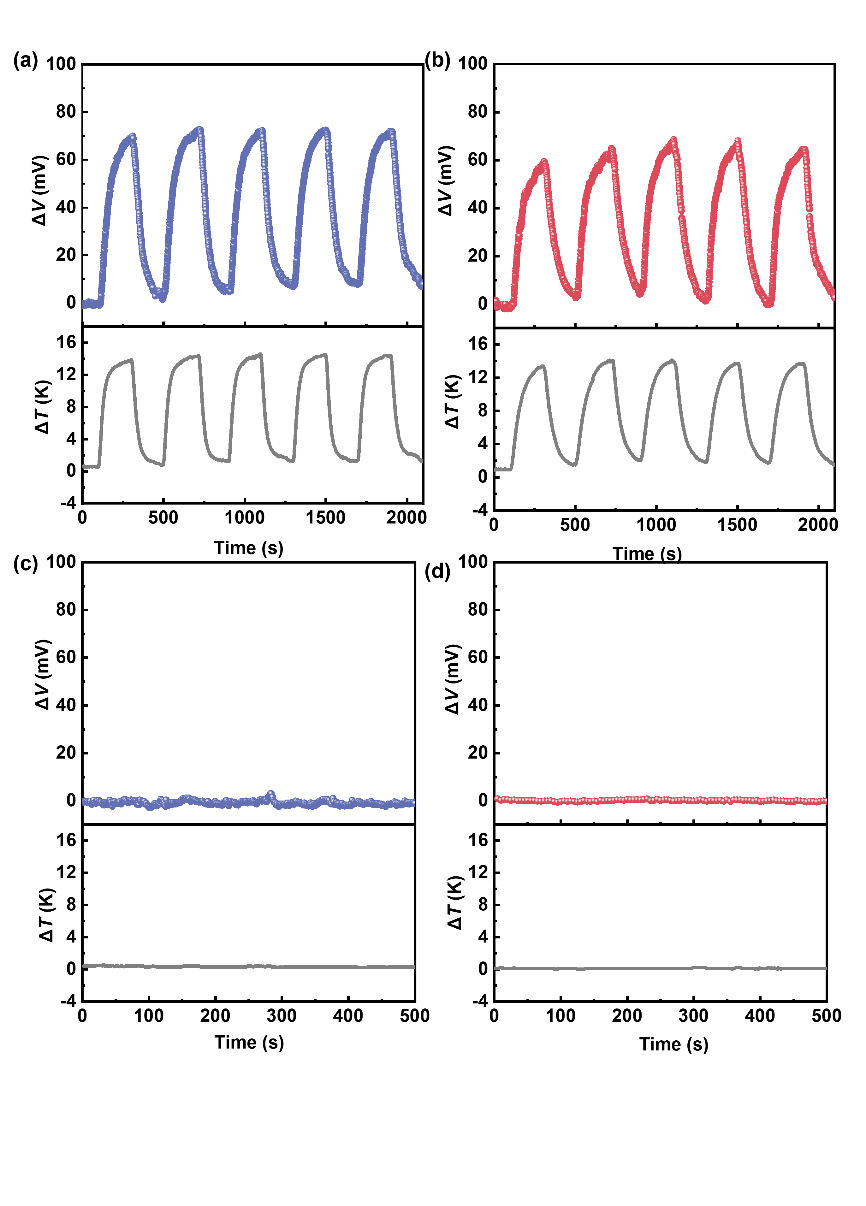


**Figure S31.** Temporal ΔV & ΔT curves for Ionogel-LT-ECl (a) in the relaxed state, and (b) under 30% strain during heating-cooling cycles. Temporal ΔV & ΔT curves of Ionogel-LT-ECl (c) in the relaxed state, and (d) under 30% strain without applying any temperature gradient.

**Table S1**. Thermoelectric properties of representative n-p convertible thermoelectric ionogel systems.

| **Ionogel Systems** | **Strategies** | **S_i_ (mV/K)** | **PF (µW m^-1^K^-2^)** | **Conditions** | **Ref.** |
| --- | --- | --- | --- | --- | --- |
| PBA/[CnMIM][NTf2] | Phase transition | -3.77 (n)  +7.71 (p) | N.A. | N.A. | 16 |
| PVDF-HFP/NaTFSI | Electrode engineering | -10.2 (n)  +20.2 (p) | N.A. | N.A. | 17 |
| PVDF-HFP/  [EMIm][TFSI](n)  PVDF-HFP/  [EMIm][TFSI]/PEG(p) | Polymer doping | -4 (n)  +14 (p) | N.A. | 70% RH | 18 |
| PVDF-HFP/  NaTFSI (p)  PVDF-HFP/  NaTFSI/TPFPB (n) | Molecular doping | -6±1 (n)  +20±4 (p) | N.A. | 68% RH | 19 |
| MA/APTA/[TFSI]^-^ (n)  MA/SPA/[EMIm]^+^ (p) | Side-chain engineering | -4.18 (n)  +5.84 (p) | 0.48 (n)  1.39 (p) | 30% RH | 20 |
| Ionogel-LT (n)  Ionogel-LT-Ecl (p) | Ionic liquid doping | -3.61 (n)  +9.74 (p) | 3.13 (n)  108.08 (p) | 40% RH | This work |

**Table S2.** The adsorption energy on the electrode surface of the n-type Ionogel-LT was calculated as: E_ads_=E_sm_-E_s_-E_m_. where Eₛₘ is the total energy of the optimized adsorption model (i.e., the (111) slab with the TFSI^-^-Li^+^ complex). Eₛ denotes the energy of the clean (111) slab, and Eₘ represents the energy of the isolated TFSI^-^-Li^+^ molecule. All energies were obtained from fully optimized geometries.

|  | Au (eV) | Ag (eV) | Cu (eV) |
| --- | --- | --- | --- |
| 111slab+TFSI^-^-Li^+^ | -313.4048 | -338.1778 | -277.9615 |
| 111slab | -224.8405 | -249.6526 | -189.5471 |
| TFSI^-^-Li^+^ | -87.8847 | -87.8847 | -87.8890 |
| **E(Adsorption Energy)** | **-0.6797** | **-0.6405** | **-0.5253** |

**Table S3.** The adsorption energy on the electrode surface of the p-type Ionogel-LT-ECl.

|  | Au (eV) | Ag (eV) | Cu (eV) |
| --- | --- | --- | --- |
| 111slab+EMIm^+^-Cl^-^ | -339.7451 | -364.3195 | -303.9913 |
| 111slab | -224.8405 | -249.6528 | -189.5471 |
| EMIm^+^-Cl^-^ | -113.7695 | -113.7565 | -113.7691 |
| **E(Adsorption Energy)** | **-1.1351** | **-0.9101** | **-0.6751** |

**Supplementary References**

1. Z. Liu, H. Cheng, Q. Le, R. Chen, J. Li, J. Ouyang, *Adv. Energy Mater.* **2022**, *12*, 2200858.
2. S. Liu, Y. Yang, H. Huang, J. Zheng, G. Liu, T. H. To, B. Huang, *Sci. Adv.* **2022**, *8*, eabj3019.
3. M. J. Abraham, T. Murtola, R. Schulz, S. Páll, J. C. Smith, B. Hess, E. Lindahl, *SoftwareX* **2015**, *1-2* 19.
4. J. Wang, P. Cieplak, P. A. Kollman, *J. Comput. Chem.* **2000**, *21*, 1049.
5. A. A. Chen, R. V. Pappu, *J. Phys. Chem. B* **2007**, *111*, 11884.
6. T. Lu, F.-W. Chen, Acta Phys. -Chim. Sin. **2012**, 28, 1.
7. B. Hess, H. Bekker, H. J. C. Berendsen, J. G. E. M. Fraaije, *J. Comput. Chem.* **1997**, *18*, 1463.
8. B. Hess, *J. Chem. Theory Comput.* **2008**, *4*, 116.
9. L. Martínez, R. Andrade, E. G. Birgin, J. M. Martínez, *J. Comput. Chem.* **2009**, *30*, 2157.
10. H. A. Posch, W. G. Hoover, F. J. Vesely, *Phys. Rev. A* **1986**, *33*, 4253.
11. T. Darden, D. York, L. Pedersen, *J. Phys. Chem.* **1993**, *98*, 10089.
12. U. Essmann, L. Perera, M. L. Berkowitz, T. Darden, H. Lee, L. G. Pedersen, *The J. Phys. Chem.* **1995**, *103*, 8577.
13. G. Kresse, D. Joubert, *Phys. Rev. B* **1999**, *59*, 1758.
14. J. P. Perdew, K. Burke, M. Ernzerhof, *Phys. Rev. Lett.* **1996**, *77*, 3865.
15. S. Grimme, J. Antony, S. Ehrlich, H. Krieg, *J. Chem. Phys.* **2010**, *132*, 154104.
16. L. Chen, C. Zhao, X. Fu, L. Zhang, X. Duan, T. Zhao, X. Zhu, M. Liu, *Mater. Horiz.* **2025**, DOI: 10.1039/D5MH01280E.
17. C. Chi, G. Liu, M. An, Y. Zhang, D. Song, X. Qi, C. Zhao, Z. Wang, Y. Du, Z. Lin, Y. Lu, H. Huang, Y. Li, C. Lin, W. Ma, B. Huang, X. Du, X. Zhang, *Nat. Commun.* **2023**, *14*, 306.
18. D. Zhao, A. Martinelli, A. Willfahrt, T. Fischer, D. Bernin, Z. U. Khan, M. Shahi, J. Brill, M. P. Jonsson, S. Fabiano, *Nat. Commun.* **2019**, *10*, 1093.
19. C. Chi, M. An, X. Qi, Y. Li, R. Zhang, G. Liu, C. Lin, H. Huang, H. Dang, B. Demir, *Nat. Commun.* **2022**, *13*, 221.
20. S. Kim, M. Ham, J. Lee, J. Kim, H. Lee, T. Park, *Adv. Funct. Mater.* **2023**, *33*, 2305499.
